# Hidden in plain sight: discovery of sand flies in Singapore and description of four species new to science

**DOI:** 10.1186/s13071-025-07021-5

**Published:** 2025-10-09

**Authors:** Huicong Ding, Majhalia Torno, Khamsing Vongphayloth, Germaine Ng, Denise Tan, Wendy Sng, Kelvin Ho, Fano José Randrianambinintsoa, Jérôme Depaquit, Cheong Huat Tan

**Affiliations:** 1 Environmental Health Institute, National Environment Agency, 11 Biopolis Way, Singapore, 138667 Singapore; 2 Université de Reims Champagne-Ardenne, UR ESCAPE, ANSES USC PETARD, Reims, France; 3 Laboratory of Vector-Borne Diseases/Zoology, Institut Pasteur du Laos, Samsenhai Road, Ban Kao-Gnot, Sisattanak District, 3560 Vientiane, Lao PDR; 4 Animal & Veterinary Service, National Parks Board, 1 Cluny Road, Singapore, 259569 Singapore; 5 Pôle de Biologie Territoriale, Laboratoire de Parasitologie-Mycologie, Centre Hospitalo-Universitaire, 51092 Reims, France

**Keywords:** Southeast Asia, Phlebotominae, *Phlebotomus*, *Sergentomyia*, *Leishmania*, Integrative taxonomy

## Abstract

**Background:**

Phlebotomine sand flies (Diptera: Psychodidae) are tiny, blood-sucking insects that are of significant public and veterinary health importance for their role in the transmission of *Leishmania* parasites, bacteria, and arboviruses. Although sand flies have been documented in most Southeast Asian countries, there are no published records confirming their presence in Singapore. Here, we present this fauna with descriptions of new species.

**Methods:**

Sand fly species identification was confirmed using an integrative taxonomic approach that combines morphological analysis with DNA barcoding of the mitochondrial *cytochrome b (cytb)* and *cytochrome c oxidase subunit I* (COI) genes.

**Results:**

We identified eight sand fly species, including four newly described species: *Phlebotomus seowpohi* n. sp., *Sergentomyia leechingae* n. sp., *Sergentomyia gubleri* n. sp., and *Sergentomyia retrocalcarae* n. sp. Phylogenetic analyses suggest that the new *Phlebotomus* species, belonging to subgenus *Euphlebotomus*, is closely related to *Phlebotomus argentipes*, an important vector of *Leishmania donovani* from the South Asian region.

**Conclusions:**

The potential risk of leishmaniasis in Singapore is compounded by the recent detection of antibodies to *Leishmania infantum* in local free-roaming dogs. Therefore, continuous monitoring is essential to assess and manage the risk of disease agent transmission, support the development of an early warning system, and enable timely and targeted public health interventions. The findings of this study contribute to the global knowledge on sand flies and enhance our understanding of local fauna diversity and distribution.

**Graphical Abstract:**

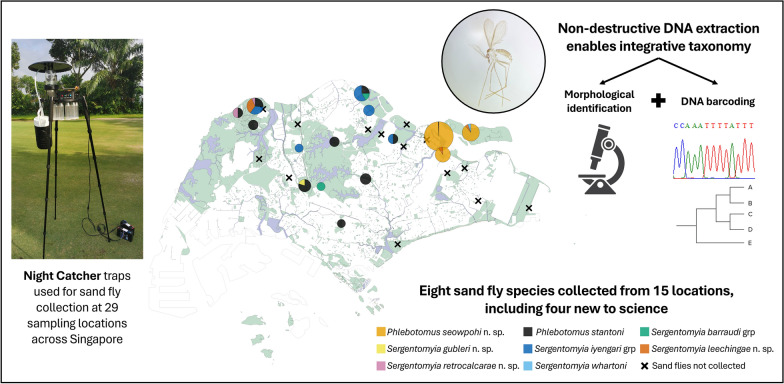

**Supplementary Information:**

The online version contains supplementary material available at 10.1186/s13071-025-07021-5.

## Background

Phlebotomine sand flies are tiny (2–3 mm in length), hematophagous insects belonging to the order Diptera, family Psychodidae. They are of significant public and veterinary health importance, with many members of this group incriminated as vectors of viral, bacterial, and parasitic diseases [1, 2]. They are widely known for their role in the transmission of *Leishmania*, an obligate, intracellular protozoan parasite, which is the causative agent of leishmaniasis, a neglected tropical disease (NTD) [3, 4].

Leishmaniasis manifests in many forms, with visceral leishmaniasis (VL) being the most significant and severe. If left untreated, VL is fatal in over 95% of cases [5]. As with most NTDs, people living in impoverished communities are disproportionately affected, with reported cases primarily concentrated in the Americas, East Africa, Middle East, and Central Asia [6]. Despite its high morbidity, the management of leishmaniasis is hindered by difficulties in managing vector populations, absence of vaccines, inaccurate disease diagnosis, and ineffective treatment options [7].

Today, 1063 valid species of sand flies have been described globally [8], with approximately 80 species incriminated as vectors of *Leishmania* species [9]. However, there is limited information about sand flies and their diversity in Southeast Asia (SEA), largely owing to the region’s long-standing history of being “leishmaniasis-free.” The detection of the first autochthonous visceral leishmaniasis case in Thailand in 1996 led to increased interest in the study of the region’s sand flies [10]. In recent years, additional reports of indigenous leishmaniasis cases due to *Leishmania martiniquensis* and *Leishmania orientalis* in different provinces of Thailand underscore the need for a comprehensive approach to understand the epidemiology of leishmaniasis, as well as the biology and diversity of sand flies in the region [11, 12].

Most research on phlebotomine sand flies in SEA has been concentrated in Thailand [13–25], with occasional surveys conducted in Cambodia [26], Indonesia [27], Laos [13, 28, 29], Malaysia [30–32], the Philippines [33–35], and Vietnam [23, 36]. Currently, the region records about 60 sand fly species belonging to the *Phlebotomus*, *Sergentomyia*, *Grassomyia*, *Idiophlebotomus*, and *Chinius* [13, 22, 37] genera. This number is likely underestimated owing to limited biosurveillance efforts in the region.

Singapore (1.3521° N, 103.8198° E) is an island state located at the southern tip of the Malay Peninsula. Despite having a tropical climate that provides an ideal habitat for sand flies [2, 38–43], to the best of our knowledge, there are no official records of phlebotomine sand flies in Singapore [8]. Here, we report their presence in Singapore for the first time. In addition, we formally describe four newly identified species belonging to the *Phlebotomus* and *Sergentomyia* genera using an integrative taxonomic approach involving morphological analyses and DNA barcoding. The findings of this study will contribute to a comprehensive global sand fly diversity checklist, which is essential for advancing sand fly research. Locally, a comprehensive understanding of sand fly diversity and distribution can provide valuable insights into the environmental factors that determine where they are most likely to thrive. This knowledge will be useful for developing risk maps to identify potential sand fly hotspots and guide the development of targeted vector control strategies to manage sand fly populations.

## Methods

### Study areas

Singapore has a total land area of 742.22 km^2^, with 49% vegetation cover [44]. The remaining 51% is of highly urbanized landscape consisting of buildings and other artificial impervious surfaces. Situated one degree north of the equator, Singapore has a tropical rainforest climate with abundant rainfall and uniformly high temperatures year-round, with no distinct seasons [45]. The average daily temperature ranges between 23 °C and 33 °C, with an average annual rainfall of 2165.9 mm.

The entomological surveillance period was from September 2020 to September 2021 at 29 sampling sites across Singapore (Additional File 1: Supplementary Fig. S1). These sites are part of Singapore’s One Health biosurveillance effort for vector-borne diseases and included secondary-growth forests, public parks, fringes of nature reserves, peri-urban vegetated areas, open fields, and coastal and urban areas.

### Sand fly collection

**Fig. 1:**
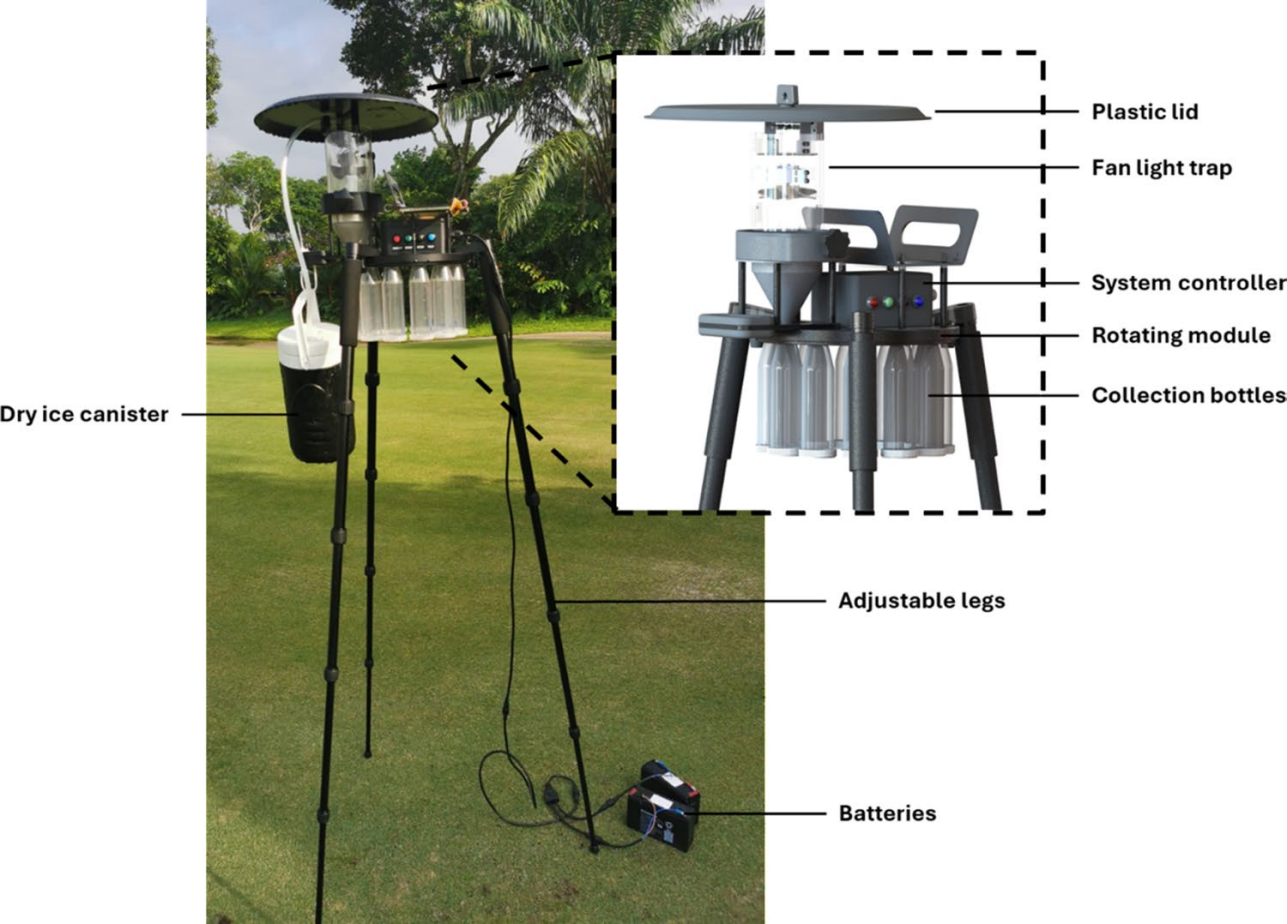
Night Catcher trap deployed for sand fly collection

The specimens were collected by the surveillance team from the National Environment Agency (NEA) as part of routine vector surveillance operations conducted under NEA’s statutory authority granted by the Control of Vectors and Pesticides Act (1998). As the collection was carried out by authorized NEA officers acting within their official capacity, no additional permits were necessary. Night Catcher (NC) traps (Orinno Technology Pte. Ltd., Singapore) were used to collect the sand flies (Fig. 1). These traps are modified Centers for Disease Control and Prevention (CDC) light traps with the additional feature of a rotating module that separates collections at hourly intervals from 1900 to 0700 h, coinciding with the peak biting time of most sand flies [46, 47]. At each sampling site, two to eight NC traps were placed at least 50 m apart. Dry ice, to generate carbon dioxide, and incandescent lights were used as trap attractants. The Global Positioning System (GPS) coordinates of each trap location were recorded. Collection bottles containing captured specimens were transported to the laboratory and placed in a −20 °C freezer overnight. After freezing, specimens were sorted, and sand flies were stored in 80% ethanol prior to species identification.

### Sample processing

Specimens were initially sorted to morpho-groups under *Phlebotomus* and *Sergentomyia* using available keys for international [48–50], as well as Southeast Asian phlebotomine sand flies [23, 35, 51–53]. For each sampling location within a morpho-group, at least one male and one female specimen were subsampled for molecular analyses. The subsampled specimens were subjected to a nondestructive DNA extraction protocol and vouchered, after which morphological analyses were performed to clarify taxonomic affinities.

### Nondestructive DNA extraction

Nondestructive DNA extraction was performed individually using the DNeasy Blood and Tissue Kit (Qiagen, Hilden, Germany), preserving morphological integrity of each specimen while extracting sufficient DNA material for downstream sequencing. During this process, sand fly specimens stored in 80% ethanol were rinsed three times with ultrapure water to remove excess ethanol. To minimize damage to the specimens during rinsing, water was removed and replaced using a pipette, instead of transferring the specimens into new tubes. After rinsing, 180 µL Buffer ATL and 20 µL Proteinase K mixture were added to each specimen, followed by whole-specimen incubation at 56 °C for 1 h. No dissection or tissue homogenization was involved. After incubation, DNA extraction following the manufacturer’s standard DNA extraction protocol with minor modifications, during which genomic DNA was eluted twice—first in 50 µL Buffer AE and subsequently in 100 µL Buffer AE. The remaining sand fly carcass was rinsed once with ultrapure water to eliminate any residual buffer prior to preservation in 80% ethanol for subsequent morphological examination.

### Morphological analyses

Soft tissue was further lysed in a 10% KOH bath then bleached in Marc-André solution. Cleared specimens were subjected to a series of alcohol baths for dehydration, then mounted on microscope slides in Euparal^®^ and covered with cover slips for species identification. Visual analysis of the specimens was performed using an Olympus BX61 compound microscope (Olympus, Japan). Quantitative measurements and counts were conducted using the Stream Motion software (Olympus, Japan) in conjunction with a video camera mounted on the microscope. For measurements of curved structures, the “polygonal line” tracing tool was used to ensure optimal accuracy. Illustrations were produced utilizing an Olympus camera lucida.

Specimens were analyzed using the previously mentioned available keys. The new species were described through an integrative taxonomic approach using morphological and molecular methods. The terminologies and abbreviations adopted for the morphological characters of phlebotomine sand flies follow that of Galati et al. [54].

### Molecular analyses

Polymerase chain reaction (PCR) amplification was performed in a 50 µL volume using 5 µL of extracted DNA and 50 pmol each of forward and reverse primers. The PCR mix contained (final concentrations): 10 mM Tris HCl (pH 8.3), 1.5 mM MgCl2, 50 mM KCl, 0.01% Triton X-100, 200 µM dNTP for each base, and 1.25 units of 5′ Taq polymerase (Eppendorf, Germany). PCR amplification was performed using the primers C3B-PDR (5′-CAYATTCAACCWGAATGATA-3′) and N1N-PDR (5′-GGTAYWTTGCCTCGAWTTCGWTATGA-3′) for *cytb* [55], and LCO1490 (5′- GGTCAACAAATCATAAAGATATTGG-3′) and HCO2198 (5′-TAAACTTCAGGGTGACCAAAAAATCA-3′) for *COI* gene fragments [56]. These amplifications were performed according to previously published conditions [55, 56]. Positive and negative controls were included for each amplification. Amplicons were purified using the E-Gel Clonewell II system (Invitrogen, USA) and subjected to bidirectional sequencing using the same primers.

Molecular analyses were performed on consensus *cytb* and *COI* sequences, respectively. Multiple alignments of consensus sequences were carried out using the ClustalW algorithm [57] in MEGA 11 software [58]. This was followed by the construction of a maximum likelihood (ML) tree with 100 bootstrap replicates. The construction was based on the substitution model selected by ModelTest [59] with an Akaike information criterion (AIC) of HKY85 [60]. Available *cytb* and *COI* sequences belonging to other known sand fly species were retrieved from GenBank [61] and included in the construction of the ML tree. Inter- and intra-specific pairwise distances were also calculated using MEGA 11 [58].

## Results

### Inventory

**Fig. 2:**
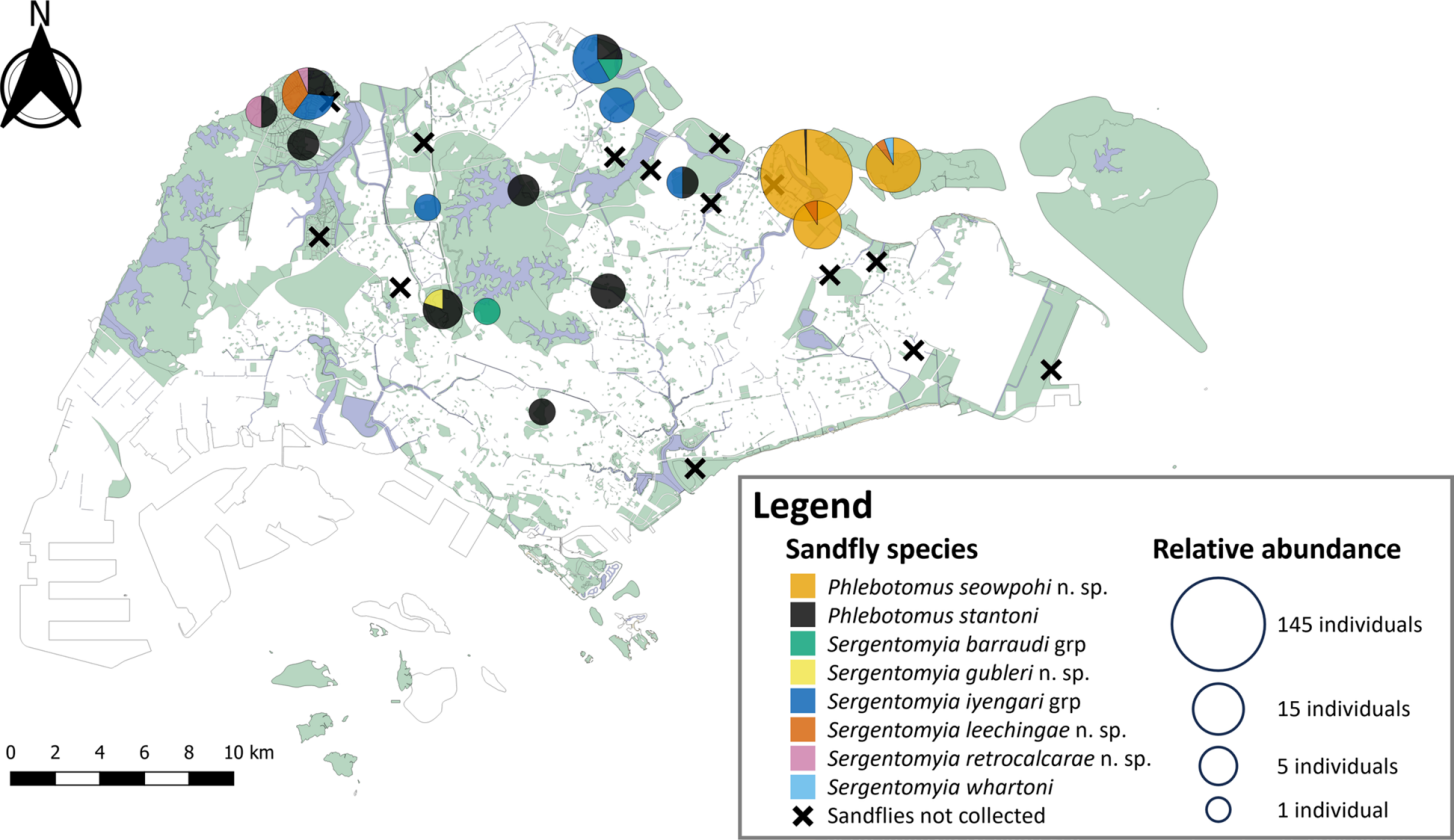
Sand fly species composition across 15 positive sampling sites in Singapore. Pie charts are scaled to the square root of the total number of individuals caught at each sampling site. The 14 sampling sites where no sand flies were collected during this study are indicated with an “×”. A total of 29 sampling sites were sampled in this study

A total of 223 phlebotomine sand flies were collected over a 1-year period (Table 1). Sand flies were collected at 15 of the 29 sampling sites (Fig. 2). Of these, 127 (57%) were identified as females, and the remaining 43% were males. The collected sand flies represented eight species across two genera, *Phlebotomus* (*n* = 192, 86.1%) and *Sergentomyia* (*n* = 31, 13.9%) (Table 1). The majority (*n* = 170, 76.2%) of the specimens caught were identified as *Phlebotomus seowpohi* n. sp., but their distribution appears to be limited to the coastal regions of northeastern Singapore (Fig. 2).

**Table 1:** Sand fly species collected in Singapore from September 2020 to September 2021; specimen voucher numbers and Genbank accession numbers for *cytb* and *COI* sequences generated in this study are listed

Genus	Subgenus	Species	Total number of specimens collected	Sampling site(s)	Number of specimens collected at each sampling site		Voucher no.	Sex	Genbank accession no.	
					F	M			*COI*	*cytb*
*Phlebotomus*	*Anaphlebotomus*	*Ph. stantoni*	22	BAMK	2	1	NEA0276 NEA0507	♂ ♀	PQ468894 PQ468898	PQ894485 PQ894487
				BG	1	0	NEA1158.5.1	♀	PQ468903	PQ894492
				CI	1	0	NEA0031	♀	PQ468888	PQ894479
				DF	3	1	NEA0079 NEA0081 NEA0569	♂ ♀ ♀	PQ468890 PQ468891 PQ468900	PQ894481 PQ894482 PQ894489
				JB	0	1	NEA0007	♂	PQ468886	PQ894477
				JS	1	2	NEA0009.1 NEA0140 NEA0839.0.1	♂ ♀ ♂	PQ468887 PQ468893 PQ468901	PQ894478 PQ894484 PQ894490
				LCK	4	0	NEA0119 NEA1015.4.1	♀ ♀	PQ468892 PQ468902	PQ894483 PQ894491
				MT	1	1	NEA0365.1 NEA0365.2	♀ ♂	PQ468896 PQ468897	PQ151959 PQ151960
				NT	2	0	NEA0330 NEA0552	♀ ♀	PQ468895 PQ468899	PQ894486 PQ894488
				PI	0	1	NEA0059	♂	PQ468889	PQ894480
	*Euphlebotomus*	*Ph. seowpohi* n. sp.	170	CI	84	60	NEA0149.1^@^ NEA0149.3^@^ NEA0149.4^*^ NEA0149.7^@^ NEA0249.1 NEA0249.4^@^ NEA0388 NEA0389.2^@^ NEA0389.3 NEA0389.7^@^ NEA0389.8 NEA0438.1 NEA0438.2 NEA0440.1 NEA0530^@^ NEA0531 NEA0951.6.1 NEA0951.6.3	♀ ♀ ♀ ♂ ♂ ♀ ♀ ♀ ♂ ♂ ♀ ♀ ♂ ♂ ♀ ♂ ♂ ♀	PQ468912 PQ468913 PQ468914 PQ468915 PQ468917 PQ468918 PQ468919 PQ468920 PQ468921 PQ468922 PQ468923 PQ468924 PQ468925 PQ468926 PQ468928 PQ468929 PQ468930 PQ468931	PQ151944 PQ151945 PQ151946 PQ151947 PQ894499 PQ151949 PQ894500 PQ151950 PQ151951 PQ151952 PQ151953 PQ894501 PQ894502 PQ151954 PQ151956 PQ894503 PQ151957 PQ151958
				PRSS	3	7	NEA0030^@^ NEA0248.4^@^ NEA0509^@^	♂ ♂ ♀	PQ468904 PQ468916 PQ468927	PQ151942 PQ151948 PQ151955
				PU	2	14	NEA0066.2 NEA0068 NEA0069.2 NEA0070 NEA0075.1 NEA0075.5 NEA0076.1^@^	♀ ♂ ♂ ♂ ♂ ♀ ♂	PQ468905 PQ468906 PQ468907 PQ468908 PQ468909 PQ468910 PQ468911	PQ894493 PQ894494 PQ894495 PQ894496 PQ894497 PQ894498 PQ151943
*Sergentomyia*	*Neophlebotomus*	*Se. iyengari* grp	17	JKM	1	0	NEA0283	♀	PQ468944	PQ151968
				JS	5	2	NEA0011.1 NEA0011.2 NEA0141 NEA0697.0.1	♀ ♂ ♀ ♀	PQ468934 PQ468935 PQ468943 PQ468949	PQ151964 PQ151965 PQ151967 PQ151970
				LCK	4	1	NEA0320 NEA0798.0.1	♂ ♀	PQ468946 PQ468951	PQ151969 PQ151972
				PI	1	0	NEA0978.1.1	♀	PQ468952	PQ894506
				YA	2	1	NEA0046.1 NEA0046.2 NEA0781.0.1	♀ ♂ ♀	PQ468936 PQ468937 PQ468950	PQ151966 PQ894505 PQ151971
	*Parrotomyia*	*Se. barraudi* grp	3	JS	1	1	NEA0009.2 NEA0501.1	♂ ♀	PQ468933 PQ468947	PQ151961 PQ151962
				RR	0	1	NEA0136	♂	PQ468942	NIL^&^
	*Rondanomyia*	*Se. whartoni*	1	PU	1	0	NEA0069.1	♀	PQ468938	PQ151977
	Ungrouped	*Se. gubleri* n. sp.	1	DF	1	0	NEA1119.7.BF1^*^	♀	PQ468953	PQ151963
		*Se. leechingae* n. sp.	7	LCK	3	2	NEA0118.1^*^ NEA0118.4^@^	♀ ♂	PQ468939 PQ468940	PQ151973 PQ151974
				PRSS	1	0	NEA0299^@^	♀	PQ468945	PQ151975
				PU	1	0	NEA0625.0.1	♀	PQ468948	NIL^&^
		*Se. retrocalcarae* n. sp.	2	JB	1	0	NEA0008^@^	♀	PQ468932	PQ894504
				LCK	1	0	NEA0120.1^*^	♀	PQ468941	PQ151976
		Total	223		127	96				

*BAMK* Bishan-Ang Mo Kio Park, *BG* Botanic Gardens, *CI* Coney Island, *DF* Dairy Farm Park, *JB* Jalan Bahtera, *JKM* Jalan Kwok Min, *JS* Jalan Selimang, *LCK* Lim Chu Kang, *MT* Mandai Track 7, *NT* Neo Tiew Lane, *PRSS* Pasir Ris-Sungei Serangoon, *PI* Picadilly, *PU* Pulau Ubin, *RR* Rifle Range Road, YA Yishun Avenue 8, *grp* group

^*^Holotype specimen

^@^Paratype specimen

^&^Unable to obtain *cytb* barcode

### Molecular analyses of phlebotomine specimens

**Fig. 3:**
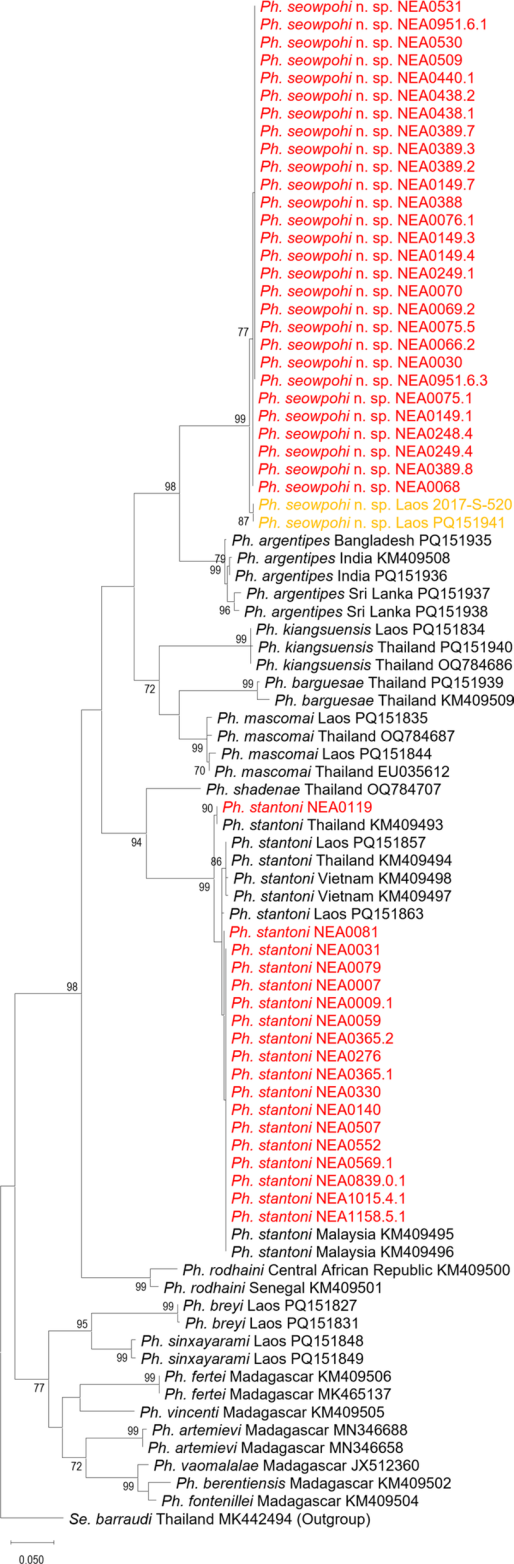
Maximum-likelihood *Phlebotomus* phylogenetic tree inferred from aligned consensus *cytb* sequences using the Hasegawa–Kishino–Yano 85 model. Sequences highlighted in red are generated from sand fly specimens collected in Singapore. Sequences in yellow are from Khammouane and Luangphabang, Laos, as part of IP Laos collection program. They will be further detailed in the discussion section. The trees have been rooted on a reference *Sergentomyia barraudi* sequence that is selected as an outgroup. Nodes with bootstrap value less than 70% are not shown

**Fig. 4:**
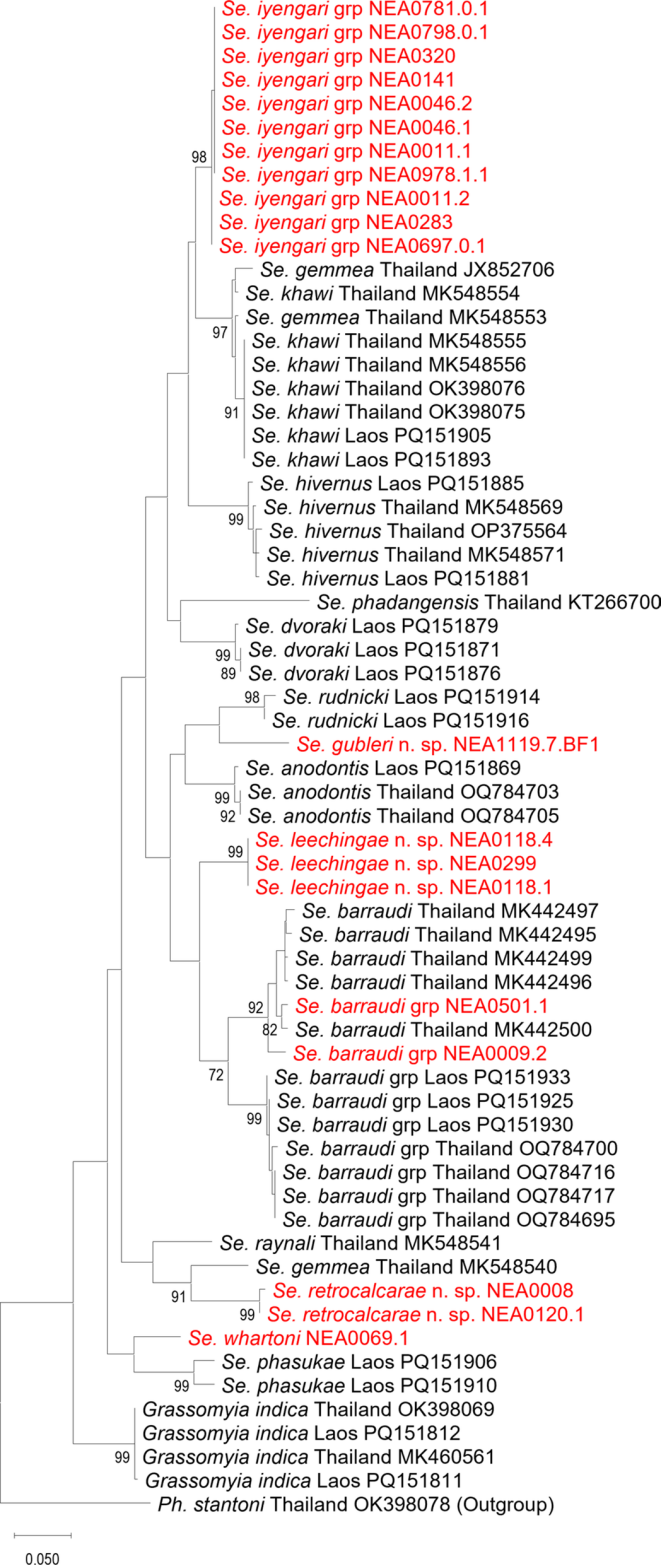
Maximum-likelihood *Sergentomyia* phylogenetic tree inferred from aligned consensus *cytb* sequences using the Hasegawa–Kishino–Yano 85 model. Sequences highlighted in red are generated from sand fly specimens collected in Singapore. The trees have been rooted on a reference *Phlebotomus stantoni* sequence that is selected as an outgroup. Nodes with bootstrap value less than 70% are not shown

In total, 68 sand fly specimens were successfully barcoded (Table 1), with 66 specimens having both their *cytb* and *COI* gene fragments amplified and sequenced. Consensus *cytb* sequences were 411 base pairs (bp) and 410 bp for *Phlebotomus* and *Sergentomyia* specimens, respectively. These consensus sequences were used to generate ML phylogenetic trees for *Phlebotomus* (Fig. 3) and *Sergentomyia* (Fig. 4) species. Sand fly species collected in Singapore formed clades with high bootstrap support (> 92%), except for the single sequences representing *Sergentomyia gubleri* n. sp. and *Sergentomyia whartoni*. Intraspecific *cytb* p-distance was less than 3.3% for species found in Singapore, while interspecific p-distance ranged from 12.5% to 21.3% when compared with reference barcodes. The calculations of p-distance over sequence pairs between and within *Phlebotomus* and *Sergentomyia* species are provided in Additional File 14: Supplementary Table S1 and Additional File 15: Supplementary Table S2, respectively.

Similarly, *COI* sequences generated were aligned and trimmed to a final length of 493 bp and 536 bp for *Phlebotomus* and *Sergentomyia* specimens, respectively. Results of molecular analyses on consensus *COI* sequences were comparable to those of the *cytb* sequences. These are provided in Additional File 2: Supplementary Fig. S2, Additional File 16: Supplementary Table S3, and Additional File 17: Supplementary Table S4.

### Description of new species

**Fig. 5:**
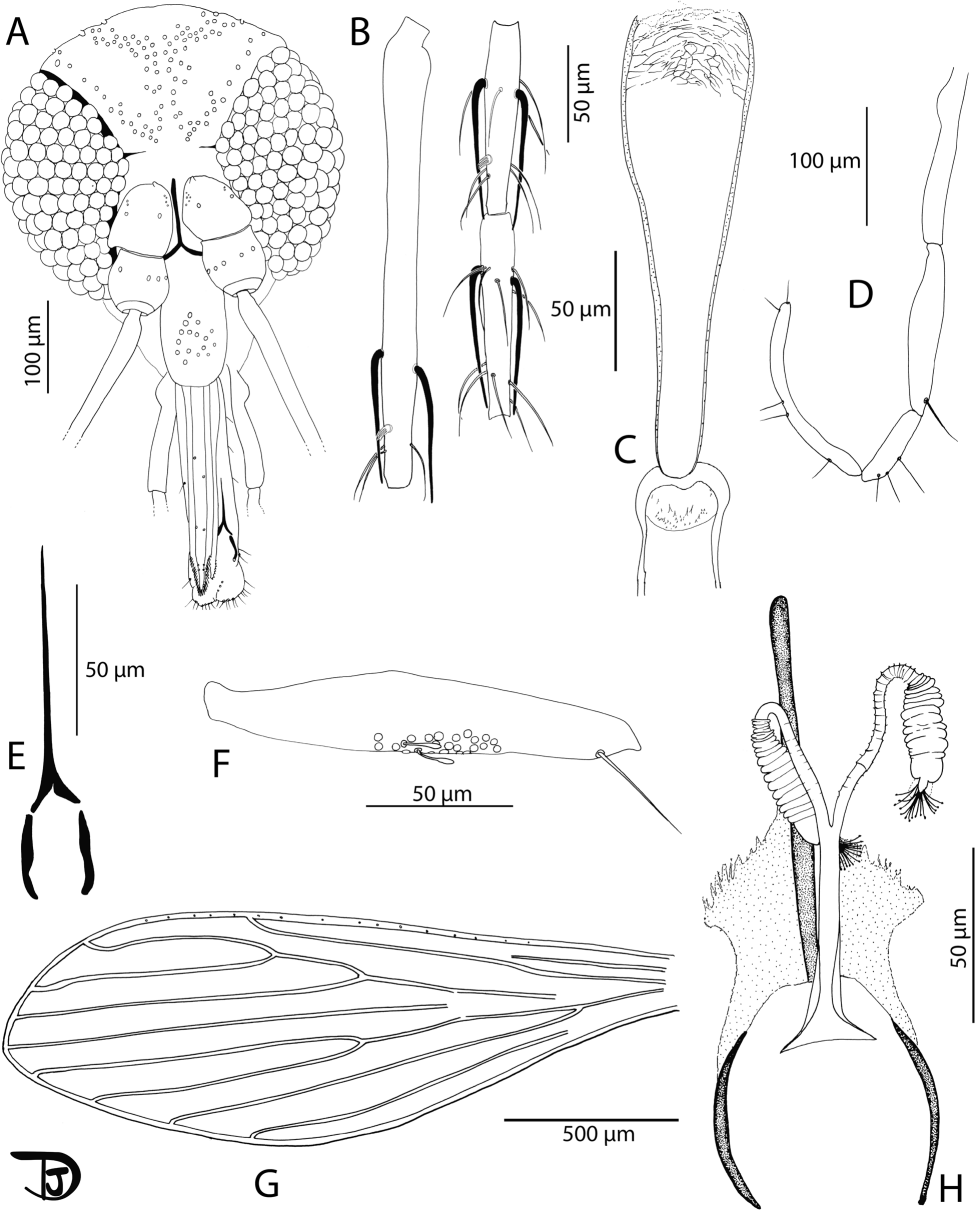
*Phlebotomus seowpohi* n. sp. female. **A** Head; **B** flagellomeres 1, 2, and 3; **C** pharynx and cibarium; **D** palpi; **E** labial furca; **F** 3rd palpal article; **G** wing; **H** genital furca and spermathecae

**Fig. 6:**
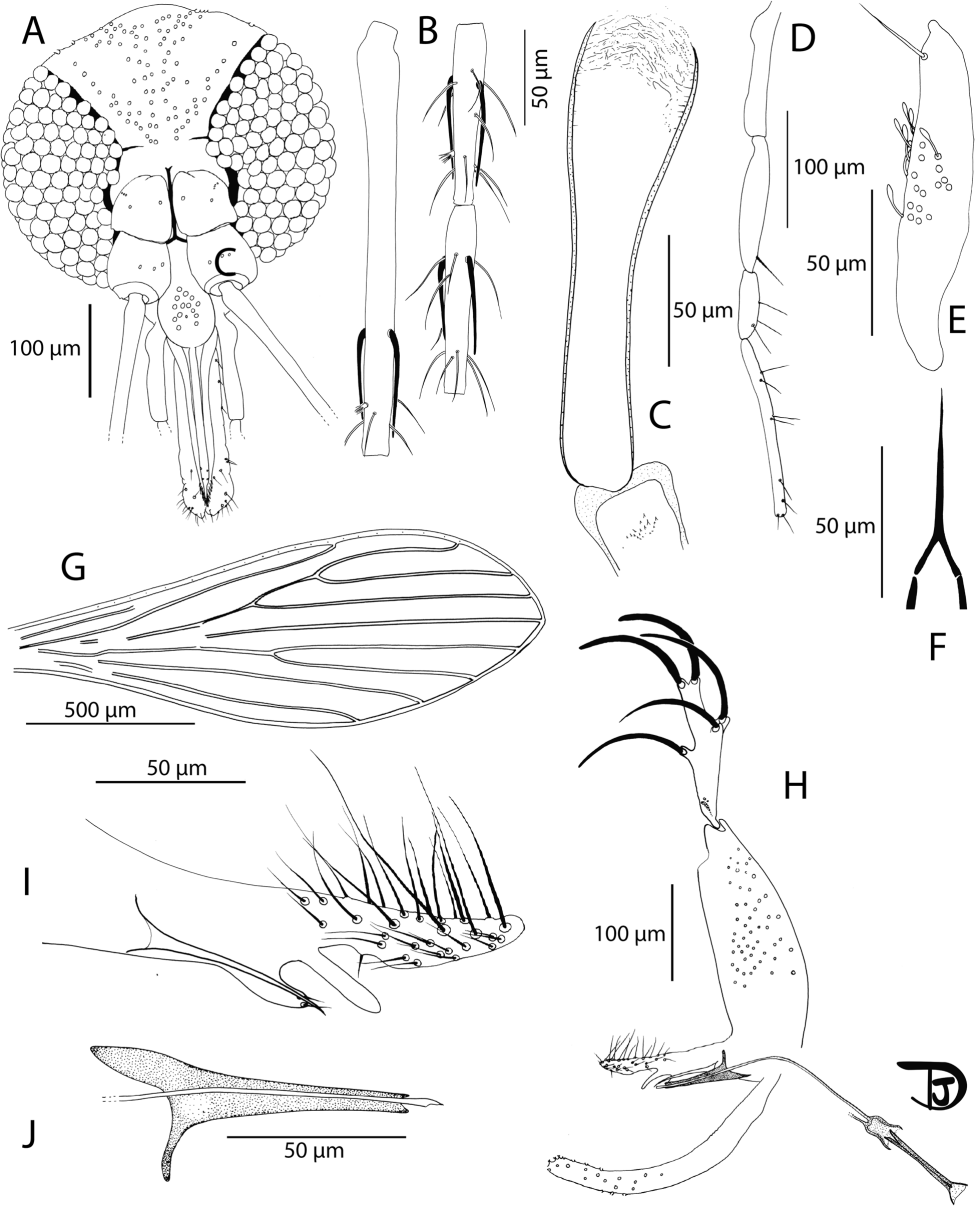
*Phlebotomus seowpohi* n. sp. male. **A** Head; **B** flagellomeres 1, 2, and 3; **C** pharynx and cibarium; **D** palpi; **E** 3rd palpal article; **F** labial furca; **G** wing; **H** genitalia; **I** trifurcated parameter and the accessory stick; **J** parameral sheath and tip of the aedeagal ducts

*Phlebotomus* (*Euphlebotomus*) *seowpohi* n. sp. Depaquit, Vongphayloth, and Tan (Figs. 5 and 6)

Family: Psychodidae Newman, 1834

Genus: *Phlebotomus* Rondani and Berté, in Rondani 1840

Subgenus: *Euphlebotomus* Theodor, 1948

Type locality: Coney Island (1°24′31.5″N, 103°55′20.9″E), Singapore.

Type material: Female holotype (voucher NEA0149.4), two female paratypes (voucher NEA0249.4 and NEA0530), and two male paratypes (voucher NEA0149.7 and NEA0030) will be deposited at the Laboratory of Entomology, The Natural History Museum, London, UK. Three female paratypes (voucher NEA0149.1, NEA0149.3, and NEA0509) and two male paratypes (voucher NEA0076.1 and NEA0248.4) will be deposited at the Environmental Health Institute, National Environment Agency, Singapore. One female paratype (voucher NEA0389.2) and one male paratype (voucher NEA0389.7) will be deposited at the Lee Kong Chian Natural History Museum, Singapore.

ZooBank registration: To comply with the regulations set out in Article 8.5 of the amended 2012 version of the International Code of Zoological Nomenclature (ICZN), details of the new species have been submitted to ZooBank. The Life Science Identifier (LSID) of the article is urn:lsid:zoobank.org:pub:13CA67C4-CFC3-437E-95F4-60BBD0F66DAD. The LSID for the new species *Phlebotomus* (*Euphlebotomus*) *seowpohi* is urn:lsid:zoobank.org:act:E9AEC641-4EC5-4740-9733-2B6CD86122AB.

Etymology: The epithet *seowpohi* refers to Mr. Seow-Poh Khoo, former Director General and Deputy Chief Executive Officer of the National Environment Agency (NEA) Singapore, in recognition of his significant contributions to improve Singapore’s public health, including prevention and control of vector-borne diseases.

To meet the criteria of availability, the authors Depaquit, Vongphayloth, and Tan are responsible for the name *Phlebotomus* *seowpohi* n. sp. and should be cited as the sole authority of this taxon, according to Article 50 (1) of the International Code of Zoological Nomenclature.

Diagnosis: coloration brown, with a dark brown scutum, in both sexes. It can be differentiated from *Ph. argentipes*, the most closely related species, by its longer ascoids.

Description and measurements indicated are those of the female holotype labeled NEA0149.4 and male paratype NEA0149.7. Measurements carried out on several specimens are indicated in Additional File 18: Supplementary Table S5.

Description

Female: Head (Fig. 5A): occiput with two narrow lines of well-individualized setae. Clypeus: 155 µm long, 75 µm wide, exhibiting 11 setae. Eyes: 270 µm long, 145 µm wide, with about 120 facets. Incomplete interocular sutures. Flagellomeres (Fig. 5B): f1 (= AIII) = 247 µm, f2 (= AIV) = 106 µm, f3 (= AV) = 104 µm. Flagellomere 1 longer than f2 + f3. Presence of two long ascoids from f1 to f13, not reaching the next articulation, except those of f1 (observed on paratype NEA0149.3). Ascoid (Fig. 5B) of f2: 74.8 µm long. Length of this ascoid/length of f2 = 0.71. One distal papilla on f1 and f2. Absence of papillae on f3 to f8. Two papillae on f9, four on f10, three on f11, five on f12 and f13, and four on f14. Several simple setae on all flagellomeres. Cibarium (Fig. 5C) armed with 5–6 anterior teeth pointed backward with 30 lateral teeth. Absence of sclerotized area. Pharyngeal armature without pigmentation, occupying the posterior fifth portion of the pharynx, including dot-like teeth and lines (Additional File 3: Supplementary Fig. S3A). A light pigmentation is observed throughout the pharynx. Palps (Fig. 5D): p1 = 35 µm, p2 = 104 µm, p3 = 147 µm, p4 = 69 µm, p5 = 186 µm. Palpal formula: 1, 4, 2, 3, 5. Presence of one distal spiniform seta on p3, four on p4, and six on p5. Presence of about 20 club-like Newstead sensilla in a medial position on P3. Absence of Newstead sensilla on the other palpal articles (Fig. 5F). Labrum: 256 µm long. f1/labrum = 0.96. Lateral teeth of the hypopharynx are not observable. Teeth of the maxillary lacinia absent. Labial furca closed (Fig. 5E).

Cervix: two cervical sensilla. Ventro-cervical sensilla not observable (paratype NEA0149.3). Thorax: 590 µm long. Brown sclerites. Mesonotum: presence of one post-alar seta; presence of three proepimeral setae; absence of the upper anepisiternal and lower anapisternal, anepimeral, metaepisternal, and metaepimeral setae; absence of setae on the anterior region of the katepisternum; absence of suture between metaepisternum and katepisternum. Metafurca mounted in lateral view on all specimens. Wings (Fig. 5G): length = 2056 μm; width = 661 μm; r5 = 1196 μm; α (r2) = 553 μm; β (r2 + 3) = 230 μm; δ = 87 μm; γ (r2 + 3 + 4) = 175 μm; ε (r3) = 684 μm; θ (r4) = 999 μm; π = 55 μm. Width/γ = 3.78. Anterior leg: procoxa = 345 µm; femur = 902 µm; tibia = 1082 µm; tarsomere I = 648 µm; sum of tarsomeres II–V = 699 µm.

Abdomen: tergites II–VI: presence of randomly distributed setae. Lack of setae on tergite VIII. Tergite IX without any protuberance. Cerci: 141 μm long. Setae not observed on sternite X. Genitalia (Fig. 5H): Spermathecal common duct 60 µm long with thicker walls roughly toward the basal 30-µm end. Opening in the genital atrium is enlarged (triangular shaped). Common duct 6–7 µm wide at its middle and 22 µm wide at its basal opening. Spermathecal individual ducts 59 μm long, 3–4 µm wide, with narrow thin wall exhibiting about 20 segments. Spermathecae (Additional File 3: Supplementary Fig. S3B): 41–46 µm long and about 12–13 µm wide. They are annealed with 15 rings. The terminal ring is larger and narrower (8–9 µm × 8–9 µm) than the other rings. Sessile head.

Male: Head (Fig. 6A): occiput with two lines of well-individualized setae. Clypeus: 122 µm long, 60 µm wide, with 13 big randomly distributed setae. Eyes: 245 µm long, 123 µm wide, with about 130 facets. Interocular sutures incomplete. Interantennal sutures do not reach the interocular sutures. Flagellomeres (Fig. 6B): f1 (= AIII) = 227 µm, f2 (= AIV) = 100 µm, f3 (= AV) = 100 µm, f12 (= AXIV) = 75 µm, f13 (= AXV) = 71 µm, f14 (= AXVI) = 80 µm. Flagellomere 1 longer than f2 + f3. Presence of two ascoids from f1 to f8, and one from f9 to f13. Ascoidal formula: 2/f1–f18, 1/f9–f13. Long ascoids not reaching the next articulation. Ascoid of f2: 60.61 µm long. Length of this ascoid/length of f2 = 0.61. One distal papilla on f1 and f2. Absence of papillae on f3 to f8. Two papillae on f9, five on f10 and f11, six on f12 and f13, and five on f14. Several simple setae on all flagellomeres. Cibarium (Fig. 6C and Additional File 4: Supplementary Fig. S4B): presence of a few small teeth (denticles) in the center. Absence of a sclerotized area. Pharyngeal armature occupying the posterior fifth portion of the pharynx with small dot-like or line-like teeth arranged concentrically (Additional File 4: Supplementary Fig. S4A). Presence of a few lateral small teeth positioned anteriorly. Palps (Fig. 6D): p1 = 33 µm, p2 = 87 µm, p3 = 117 µm, p4 = 57 µm, p5 = 156 µm. Palpal formula: 1, 4, 2, 3, 5. Presence of a group of about 15 club-like Newstead’s sensilla (Fig. 6E) in the middle of the third palpal segment. No Newstead’s sensilla on other palpal segments. Presence of one distal spiniform seta on p3, three on p4, and six on p5. Labrum: 178 µm long. f1/labrum = 1.28. Labial furca closed (Fig. 6F).

Cervix: two cervical sensilla on each side. Thorax: 480 µm long. Brown sclerites. Mesonotum: presence of one post-alar seta; presence of three proepimeral setae; absence of upper anepisiternal and lower anapisternal, anepimeral, metaepisternal, and metaepimeral setae; absence of setae on the anterior region of the katepisternum; absence of suture between metaepisternum and katepimeron. Metafurca with separated vertical arms. Wings (Fig. 6G): length = 1622 μm; width = 577 μm; r5 = 1009 μm; α (r2) = 448 μm; β (r2 + 3) = 195 μm; δ = 410 μm; γ (r2 + 3 + 4) = 145 μm; ε (r3) = 580 μm; θ (r4) = 836 μm; π = 67 μm. Width/γ = 3.97. Anterior leg: procoxa = 298 µm; femur = 740 µm; tibia = 1002 µm; tarsomere I = 635 µm; sum of tarsomeres II–V = 650 µm. Median leg: mesocoxa = 329 µm; femur = 677 µm; tibia = 1164 µm; tarsomere I = 750 µm; sum of tarsomeres II–V = 663 µm. Posterior leg: metacoxa = 360 µm; femur = 772 µm; tibia = 1414 µm; tarsomere I = 831 µm; sum of tarsomeres II–V = 749 µm.

Abdomen: tergites II–VII: presence of randomly distributed setae. Presence of two tergal papillae (one per side) on tergites IV–VII. Genitalia (Fig. 6H and Additional File 4: Supplementary Fig. S4C): Absence of hypandrial apodeme (= abdominal rod). Gonocoxite: 258 µm long with a median tuft of about 30 internal setae. Absence of basal gonocoxal lobe. Gonostyle: 167 µm long with five thick spines (two terminal and three median ones). Absence of accessory setae. Parameres: complex (Fig. 6H, I), 150 µm long, with a long upper lobe (70 µm long) exhibiting many setae, a brown shorter and narrow hairless intermediate lobe (30 µm long), and a small lower lobe (22 µm long) exhibiting two setae. Presence of a long (75 µm long), pale accessory spine (Additional File 4: Supplementary Fig. S4D) between the paramere and the parameral sheath, narrow, pointed at its tip. Parameral sheath (Fig. 6J): 87 µm long, blunt end. Aedeagal ducts: 235 µm long, smooth, beveled at the tip (Fig. 6J). Sperm pump: 126 µm long with a narrow ejaculatory apodeme 102 µm long. Epandrial lobes: 300 µm long, longer than the gonocoxite, without permanent setae. Cerci: 150 μm long.

**Fig. 7:**
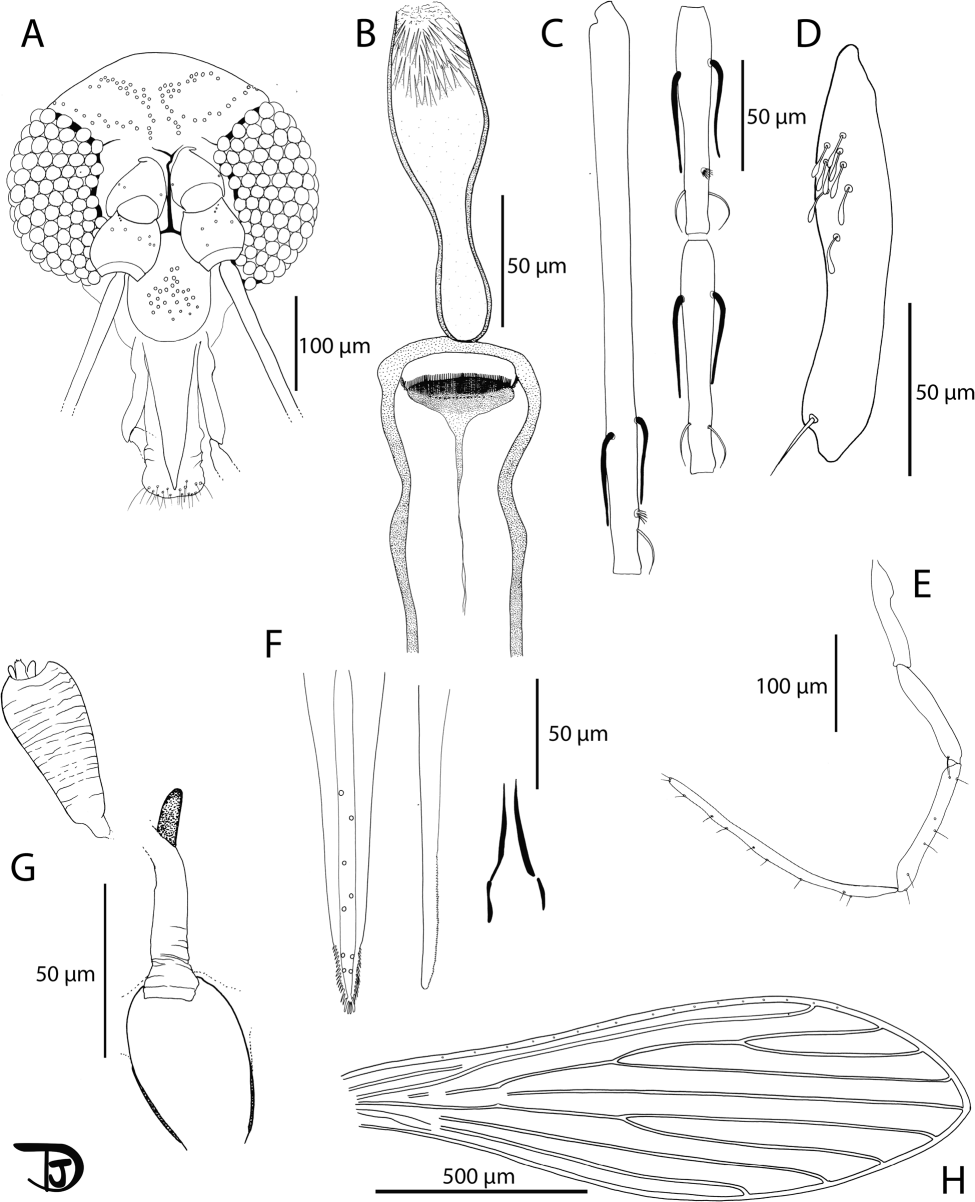
*Sergentomyia gubleri* n. sp. female. **A** Head; **B** pharynx and cibarium; **C** flagellomeres 1, 2, and 3; **D** 3rd palpal article; **E** palpi; **F** mouth parts labrum, mandible, and labial furca from left to right; **G** genital furca and spermathecae; **H** wing

*Sergentomyia gubleri* n. sp. Depaquit, Vongphayloth, and Torno (Fig. 7)

Family: Psychodidae Newman, 1834

Genus: *Sergentomyia* França and Parrot, 1920

Type locality: Dairy Farm (1°20′54.8″N, 103°46′37.5″E), Singapore.

Type material: Female holotype (voucher NEA1119.7.BF1) will be deposited at the Laboratory of Entomology, The Natural History Museum, London, UK.

ZooBank registration: To comply with the regulations set out in Article 8.5 of the amended 2012 version of the International Code of Zoological Nomenclature (ICZN), details of the new species have been submitted to ZooBank. The Life Science Identifier (LSID) of the article is urn:lsid:zoobank.org:pub:13CA67C4-CFC3-437E-95F4-60BBD0F66DAD. The LSID for the new species *Sergentomyia gubleri* is urn:lsid:zoobank.org:act:54FFAED6-04F4-48E9-8F77-1E99DECD08ED.

Etymology: The epithet *gubleri* refers to Emeritus Professor Duane J. Gubler, Duke-National University of Singapore, in recognition of his significant contributions to public health entomology. His decades of work have enhanced the understanding and control of vector-borne diseases and significantly improved public health outcomes worldwide.

To meet the criteria of availability, the authors Depaquit, Vongphayloth, and Torno are responsible for the name *Sergentomyia gubleri* n. sp. and should be cited as the sole authority of this taxon, according to Article 50 (1) of the International Code of Zoological Nomenclature.

Diagnosis: *Sergentomyia gubleri* n. sp. exhibits many cibarial comb-like teeth. It can be differentiated from *Se. rudnicki* and *Se. brevicaulis* by the length of the first flagellomere as well as by the number of cibarial teeth.

The male remains unknown. Description and measurements indicated are those of the female holotype labeled NEA1119.7.BF1.

Description

Female: Head (Fig. 7A): occiput with two narrow lines of well-individualized setae. Clypeus: 96 µm long 84 µm wide, exhibiting 25 setae. Eyes: 177 µm long, 82 µm wide, with about 110 facets. Incomplete interocular sutures. Interantennal sutures do not reach the interocular ones. Flagellomeres (Fig. 7C): f1 (= AIII) = 250 µm, f2 (= AIV) = 101 µm, f3 (= AV) = 100 µm, f12 (= AXIV) = 72 µm, f13 (= AXV) = 55 µm, f14 (= AXVI) = 40 µm. Flagellomere 1 longer than f2 + f3. Presence of 2 ascoids from f1 to f13. Ascoidal formula: 2/f1–f13. One distal papilla on f1, f2, f6, and f7. Absence of papillae on f3 to f5. Three papillae on f8 to f10 (two posterior and one median), four on f11 (two posterior, one median, and one distal), and five on f12 (two posterior, two median, and one distal). One simple seta on f1, two on f2 to f11, three on f12, and nine on f13. Palps (Fig. 7D, E): p1 = 45 µm, p2 = 67 µm, p3 = 113 µm, p4 = 139 µm, p5 = 257 µm. Palpal formula: 1, 2, 3, 4, 5. Presence of a group of about eight club-like Newstead’s sensilla in the middle of the third palpal segment (Fig. 7D). No Newstead’s sensilla on other palpal segments. Presence of one apical spiniform seta on p3, seven on p4, and about ten on p5. Labrum: 142 µm long. f1/labrum = 1.76. About 60 tiny teeth on the mandible (Fig. 7F, middle). Labrum with about 18 lateral teeth on each side (Fig. 7F, left). Teeth of the maxillary lacinia are not observable. Labial furca open (Fig. 7F, right). Cibarium (Fig. 7B and Additional File 8: Supplementary Fig. S8A) armed with 68 posterior horizontal teeth and about 15 anterior vertical teeth. Presence of a distinctly dark oval sclerotized area, not reaching the posterior tip of the horizontal teeth. Well-developed pharyngeal armature occupies the posterior third part of the pharynx with about 40 lightly pigmented, long and pointed backward oriented teeth (Fig. 7B and Additional File 8: Supplementary Fig. S8B).

Cervix: two cervical sensilla. Ventro-cervical sensilla not observable.

Thorax: brown sclerites. Mesonotum: absence of post-alar, proepimeral, upper anepisiternal, lower anapisternal, anepimeral, metaepisternal, and metaepimeral setae; absence of setae on the anterior region of the katepisternum. Metafurca with long separated vertical and horizontal arms. Wings (Fig. 7H): length = 1497 μm; width = 464 μm; r5 = 1058 μm; α (r2) = 332 μm; β (r2 + 3) = 285 μm; δ = 176 μm; γ (r2 + 3 + 4) = 262 μm; ε (r3) = 431 μm; θ (r4) = 776 μm; π = 16 μm. Width/γ = 1.77. Anterior leg: procoxa = 255 µm; femur = 560 µm; tibia = 505 µm; tarsomere I = 260 µm; sum of tarsomeres II–V = 452 µm. Median leg: mesocoxa = 262 µm; femur = 607 µm; tibia = 626 µm; tarsomere I = 290 µm; sum of tarsomeres II–V = 489 µm. Posterior leg: metacoxa = 265 µm; femur = 607 µm; tibia = 788 µm; tarsomere I = 355 µm; sum of tarsomeres II–V = 727 µm.

Abdomen: tergites II–VI: presence of randomly distributed setae. Lack of setae on tergite VIII. Tergite IX without any protuberance. Cerci: 149 μm long. Setae not observed on sternite X. Spermathecae (Fig. 7G and Additional File 8: Supplementary Fig. S8E): 52 µm long and about 26 µm wide with wrinkled thin walls. Wider at its apex than at the proximal part. Sessile head.

**Fig. 8:**
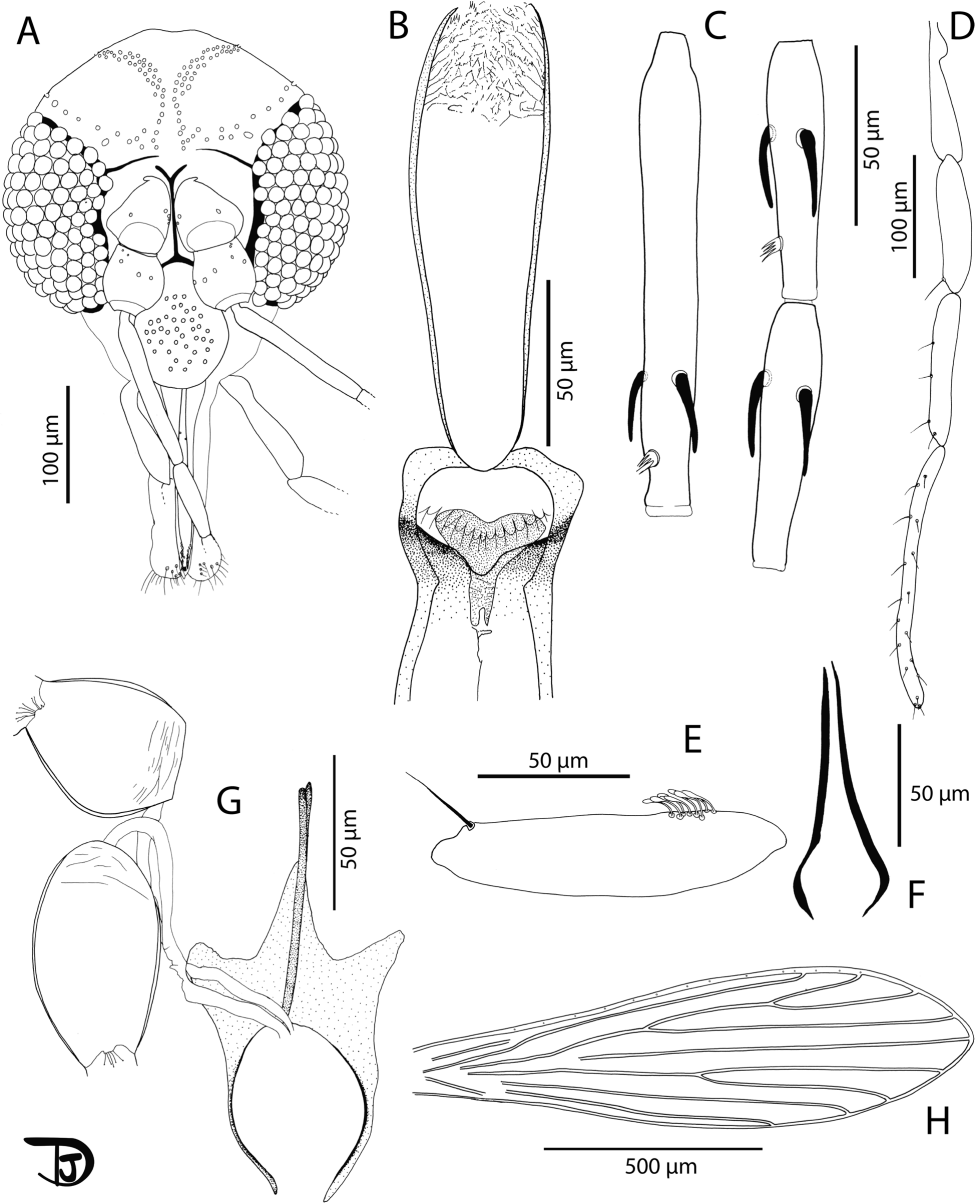
*Sergentomyia leechingae* n. sp. female.** A** Head; **B** pharynx and cibarium; **C** flagellomeres 1, 2, and 3; **D** palpi; **E** 3rd palpal article; **F** labial furca; **G** genital furca and spermathecae; **H** wing

**Fig. 9:**
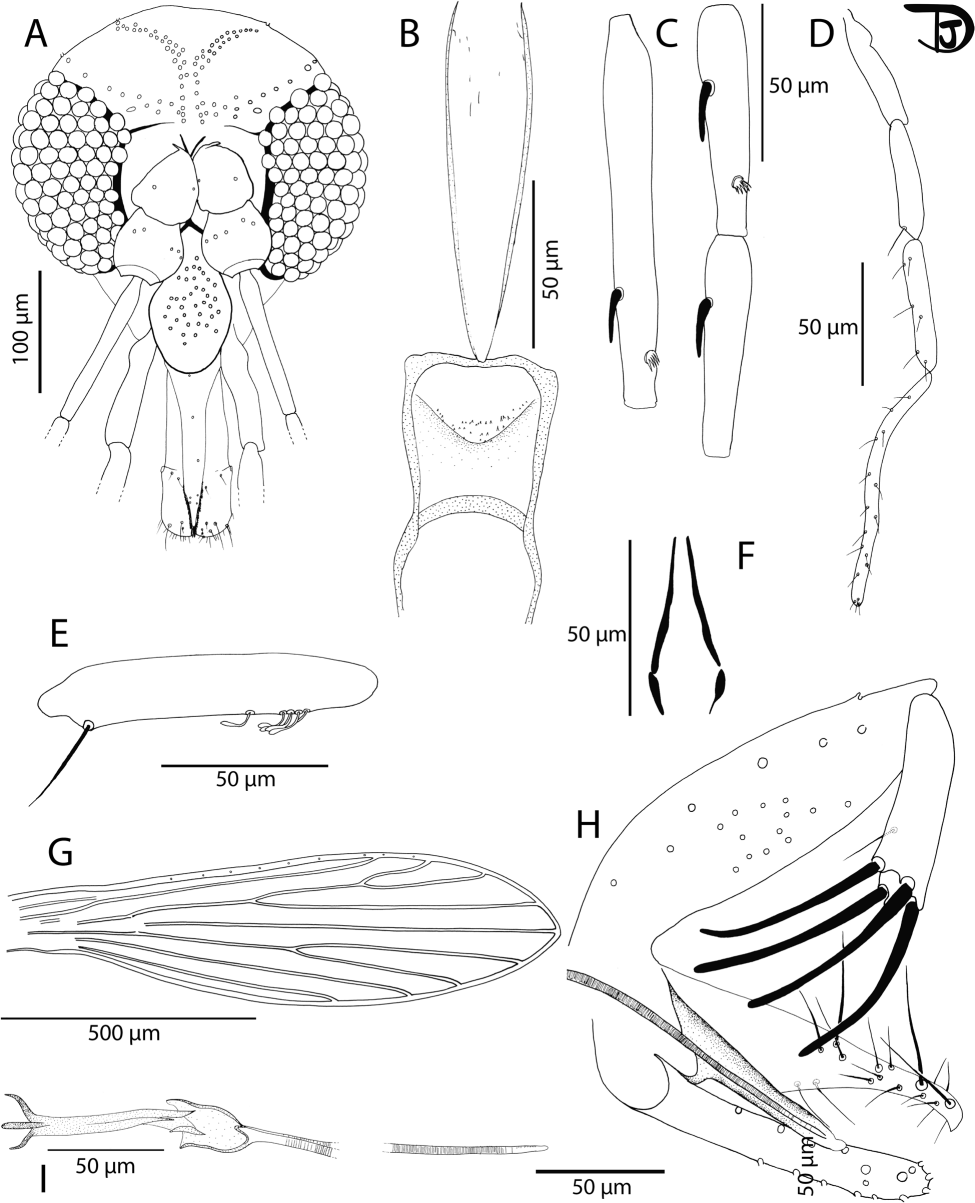
*Sergentomyia leechingae* n. sp. male. **A** Head; **B** pharynx and cibarium; **C** flagellomeres 1, 2, and 3; **D** palpi; **E** 3rd palpal article; **F** labial furca; **G** wing; **H** genitalia; **I** sperm pump and tip of the aedeagal ducts

*Sergentomyia leechingae* n. sp. Depaquit, Vongphayloth, and Ding (Figs. 8 and 9)

Family: Psychodidae Newman, 1834

Genus: *Sergentomyia* França and Parrot, 1920

Type locality: Lim Chu Kang, Singapore (1°26′12.6″N, 103°42′50.6″E).

Type material: female holotype (voucher NEA0118.1) and one male paratype (voucher NEA0118.4) will be deposited at the Laboratory of Entomology, The Natural History Museum, London, UK. One female paratype (voucher NEA0299) will be deposited at the Environmental Health Institute, National Environment Agency, Singapore.

ZooBank registration: To comply with the regulations set out in Article 8.5 of the amended 2012 version of the International Code of Zoological Nomenclature (ICZN), details of the new species have been submitted to ZooBank. The Life Science Identifier (LSID) of the article is urn:lsid:zoobank.org:pub:13CA67C4-CFC3-437E-95F4-60BBD0F66DAD. The LSID for the new species *Sergentomyia leechingae* is urn:lsid:zoobank.org:act:F43004C9-99FD-488A-903C-D63013471B06.

Etymology: The epithet *leechingae* refers to Associate Professor Lee-Ching Ng, Group Director of the Environmental Health Institute, NEA, Singapore, in recognition of her significant contributions to Singapore’s public health and her impactful work in the prevention and control of vector-borne diseases both in Singapore and globally.

To meet the criteria of availability, the authors Depaquit, Vongphayloth, and Ding are responsible for the name *Sergentomyia leechingae* n. sp. and should be cited as the sole authority of this taxon, according to Article 50 (1) of the International Code of Zoological Nomenclature.

Diagnosis: *Sergentomyia leechingae* n. sp. exhibits a cibarial notch typical of the *Se. babu* group. It can be differentiated from other members of this group by the shape of the cibarial notch and the number of teeth on the cibarial armature.

Description and measurements indicated are those of the female holotype labeled NEA0118.1. and of the male paratype NEA0118.4.

Description

Female: Head (Fig. 8A): occiput with two narrow lines of well-individualized setae. Clypeus: 118 µm long, 70 µm wide, exhibiting 39 setae. Eyes: 175 µm long, 76 µm wide, with about 100 facets. Incomplete interocular sutures. Interantennal sutures do not reach the interocular sutures. Flagellomeres (Fig. 8C): f1 (= AIII) = 125 µm, f2 (= AIV) = 70 µm, f3 (= AV) = 65 µm, f12 (= AXIV) = 45 µm, f13 (= AXV) = 39 µm, f14 (= AXVI) = 38 µm. Flagellomere 1 shorter than f2 + f3. Presence of two short ascoids from f1 to f13, not reaching the next articulation. Ascoidal formula: 2/f1–f13. One distal papilla on f1 and f2. Absence of papillae on f3 to f12. Three papillae on f12 and f13, and two on f14. Palps (Fig. 8D): p1 = 41 µm, p2 = 65 µm, p3 = 98 µm, p4 = 120 µm, p5 = 206 µm. Palpal formula: 1, 2, 3, 4, 5. Presence of a group of less than ten club-like Newstead’s sensilla in the proximal part of p3 (Fig. 8E). No Newstead’s sensilla on other palpal segments. Presence of one distal spiniform setae on p3, five on p4, and about 15 on p5. Labrum: 147 µm long. f1/labrum = 0.85. Mouth parts are not observable. Labial furca open (Fig. 8F). Cibarium armed with 14 posterior teeth pointed backward (Fig. 8B). Absence of anterior teeth. Presence of heart-shaped sclerotized area, with a bifid anterior extension. Presence of a notch in the middle of the cibarium, just below the area where the anterior teeth would have been located. Pharyngeal armature is inconspicuous, with some pointed teeth oriented backward and some tiny teeth arranged along numerous lines (Fig. 8B and Additional File 10: Supplementary Fig. S10A). Armature occupies the posterior quarter of the pharynx.

Cervix: two cervical sensilla on each side. Ventro-cervical sensilla not observed.

Thorax: 525 µm long. Light-brown sclerites. Mesonotum: absence of post-alar, proepimeral, upper anepisiternal, lower anapisternal, anepimeral, metaepisternal, and metaepimeral setae; setae on the anterior region of the katepisternum were not observed. Metafurca mounted in lateral view on all specimens. Wings (Fig. 8H): length = 1122 μm; width = 369 μm; r5 = 887 μm; α (r2) = 160 μm; β (r2 + 3) = 268 μm; δ = 39 μm; γ (r2 + 3 + 4) = 220 μm; ε (r3) = 269 μm; θ (r4) = 627 μm; π = 82 μm. Width/γ = 1.68. Anterior leg: procoxa = 235 µm; femur = 503 µm; tibia = 430 µm; tarsomere I = 220 µm; sum of tarsomeres II–V = 419 µm.

Abdomen: tergites II–VI: presence of randomly distributed setae. Lack of setae on tergite VIII. Tergite IX without any protuberance. Cerci: 90 μm long. Setae not observed on sternite X.

Absence of common duct of spermathecae (Fig. 8G). individual ducts of spermathecae are 125 µm long. Spermathecae are 57 µm long and 33 µm wide, oblong or oval with slight streaks at the base (Additional File 10: Supplementary Fig. S10B). Sessile head.

Male: head (Fig. 9A): occiput with two lines of well-individualized setae. Clypeus: 117 µm long, 63 µm wide, with 34 big randomly distributed setae. Eyes: 165 µm long, 82 µm wide, with about 90 facets. Interocular sutures incomplete, not observable. Interantennal sutures do not reach the interocular sutures. Flagellomeres (Fig. 9C): f1 (= AIII) = 116 µm, f2 (= AIV) = 69 µm, f3 (= AV) = 68 µm. Flagellomere 1 shorter than f2 + f3. Presence of one short ascoid from f1 to f7, not reaching the next articulation. Antenna is broken after f7. One distal papilla on f1 and f2. Absence of papilla on f3 to f8. Palps (Fig. 9D, E): p1 = 33 µm, p2 = 87 µm, p3 = 117 µm, p4 = 57 µm, p5 = 156 µm. Palpal formula: 1, 4, 2, 3, 5. Presence of six club-like Newstead’s sensilla at the proximal third of the palpal segment (Fig. 9E). No Newstead’s sensilla on other palpal segments. Presence of one distal spiniform seta on p3, six on p4, and about 15 on p5. Labrum: 129 µm long. f1/labrum = 0.89. Labial furca open (Fig. 9F). Cibarium (Fig. 9B and Additional File 11: Supplementary Fig. S11A): presence of a few small randomly arranged teeth (denticles) in the middle. Absence of a sclerotized area. Very few teeth observed at the posterior part of the pharynx (Fig. 9B).

Cervix: two cervical sensilla on each side. Ventro-cervical sensilla not observed.

Thorax: brown sclerites. Mesonotum: absence of post-alar, proepimeral, upper anepisiternal, lower anapisternal, anepimeral, metaepisternal, and metaepimeral setae; absence of setae on the anterior region of the katepisternum. Metafurca with separated vertical arms. Wings (Fig. 9G): length = 1030 μm; width = 292 μm; r5 = 777 μm; α (r2) = 139 μm; β (r2 + 3) = 206 μm; δ = 37 μm; γ (r2 + 3 + 4) = 178 μm; ε (r3) = 233 μm; θ (r4) = 533 μm; π = 74 μm. Width/γ = 1.64. Legs broken.

Abdomen: tergite II–VII: presence of randomly distributed setae. Presence of two tergal papillae (one per side) on tergites IV–VII. Genitalia (Fig. 9H and Additional File 11: Supplementary Fig. S11B): Gonocoxite: 173 µm long with around ten internal setae scattered randomly around the medial region. Absence of basal gonocoxal lobe. Gonostyle: 75 µm long with four thick terminal spines. Presence of an accessory seta on the distal quarter of the gonostyle. Parameres: simple, 127 µm long. Parameral sheath: 89 µm long. Aedeagal ducts: 240 µm long. Tip of the ducts rounded (Fig. 9I). Sperm pump (Fig. 9I): 96 µm long with a narrow ejaculatory apodeme, 82 µm long. Epandrial lobes: 138 µm long, shorter than the gonocoxite. Cerci: 150 μm long.

**Fig. 10:**
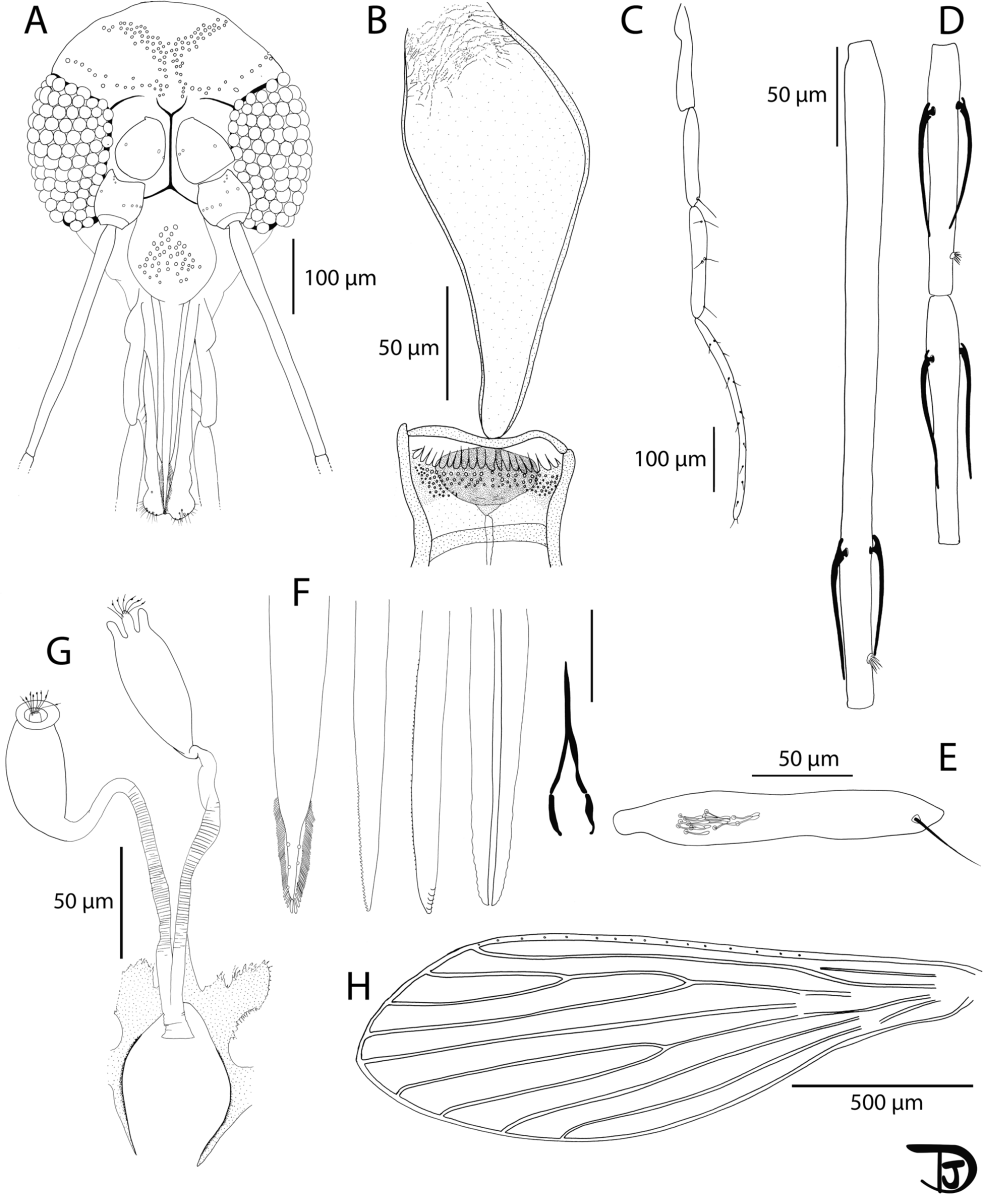
*Sergentomyia retrocalcarae* n. sp. female. **A** Head; **B** pharynx and cibarium; **C** palpi; **D** flagellomeres 1, 2, and 3; **E** 3rd palpal article; **F** mouth parts labrum, mandible, lacinia, hypopharynx, and labial furca from left to right; **G** genital furca and spermathecae; **H** wing

*Sergentomyia retrocalcarae* n. sp. Depaquit, Vongphayloth, and Torno (Fig. 10)

Family: Psychodidae Newman, 1834

Genus: *Sergentomyia* França and Parrot, 1920

Type locality: Lim Chu Kang (1°26′35.7″N, 103°42′28.6″E), Singapore.

Type material: Female holotype (voucher NEA0120.1) will be deposited at the laboratory of Entomology, The Natural History Museum, London, UK. One female paratype (voucher NEA0008) will be deposited at the Environmental Health Institute, National Environment Agency, Singapore.

ZooBank registration: To comply with the regulations set out in Article 8.5 of the amended 2012 version of the International Code of Zoological Nomenclature (ICZN), details of the new species have been submitted to ZooBank. The Life Science Identifier (LSID) of the article is urn:lsid:zoobank.org:pub:13CA67C4-CFC3-437E-95F4-60BBD0F66DAD. The LSID for the new species *Sergentomyia retrocalcarae* is urn:lsid:zoobank.org:act:362BA0ED-4924-41CE-8E24-91D80FF9E2CB.

Etymology: From Latin *retro*, oriented backward, and *calcar*, spur, referring to spurs on the ascoids, which are oriented backward.

To meet the criteria of availability, the authors Depaquit, Vongphayloth, and Torno are responsible for the name *Sergentomyia retrocalcarae* n. sp. and should be cited as the sole authority of this taxon, according to Article 50 (1) of the International Code of Zoological Nomenclature.

Diagnosis: *Sergentomyia retrocalcarae* n. sp. exhibit spurs on the ascoids that are oriented backward on the flagellomeres. Its cibarium differs from that of *Sergentomyia raynali* and it exhibits less cibarial teeth than *Se. gombaki* and *Se. arboris*.

The male remains unknown. Description and measurements indicated are those of the female holotype labeled NEA0120.1 except those of the genitalia observed on paratype NEA0008.

Description

Female: head (Fig. 10A): occiput with two narrow lines of well-individualized setae. Clypeus: 138 µm long, 65 µm wide, exhibiting 44 setae. Eyes: 205 µm long, 98 µm wide, with about 80 facets. Interocular sutures incomplete. Interantennal sutures do not reach the interocular sutures. Flagellomeres (Fig. 10D): f1 (= AIII) = 319 µm, f2 (= AIV) = 120 µm, f3 (= AV) = 119 µm, f12 (= AXIV) = 75 µm, f13 (= AXV) = 58 µm, f14 (= AXVI) = 53 µm. Flagellomere 1 longer than f2 + f3. Presence of two ascoids from f1 to f13 (Additional File 12: Supplementary Fig. S12A). Ascoidal formula: 2/f1–f13. One apical papilla on f1 and f2. Absence of papilla on f3 to f5. One papilla on f6 to f11, and four on f12 to f14. Palps (Fig. 10C, E): p1 = 57 µm, p2 = 104 µm, p3 = 149 µm, p4 = 174 µm, p5 = 306 µm. Palpal formula: 1, 2, 3, 4, 5. Presence of a dozen club-like Newstead’s sensilla located at the proximal third of the third palpal segment (Fig. 10E). No Newstead’s sensilla on other palpal segments. Presence of 1 apical spiniform seta on p3, 5 on p4, and 13 on p5. Labrum: 258 µm long. f1/labrum = 1.24. More than 50 lateral teeth on each side of the labrum (Fig. 10F, left). Mandible exhibiting about 40 lateral teeth (Fig. 10F, middle left). Hypopharynx exhibiting about ten waved teeth on each side (Fig. 10F, center). Maxillary lacinia exhibiting 5 external teeth and about 50 internal teeth (Fig. 10F, middle right). Labial furca open (Fig. 10F, right). Cibarium armed with 19 horizontal posterior horizontal teeth pointed backward (Fig. 10B). Presence of many anterior teeth. Presence of an oval sclerotized area that does not reach the lateral walls of the cibarium but reaches the tip of the horizontal teeth. Pharynx slightly pigmented under the armature. Pharyngeal armature at the posterior fifth of the pharynx, consisting of minute teeth forming concentric curves (Fig. 10B and Additional File 12: Supplementary Fig. S12B).

Cervix: two cervical sensilla. Ventro-cervical sensilla not observable.

Thorax: brown sclerites. Mesonotum: absence of post-alar, proepimeral, upper anepisiternal, lower anapisternal, anepimeral, metaepisternal, and metaepimeral setae; absence of setae on the anterior region of the katepisternum. Metafurca with long separated vertical arms. Wings (Fig. 10H): length = 1600 μm; width = 554 μm; r5 = 1196 μm; α (r2) = 348 μm; β (r2 + 3) = 329 μm; δ = 145 μm; γ (r2 + 3 + 4) = 317 μm; ε (r3) = 465 μm; θ (r4) = 860 μm; π = 87 μm. Width/γ = 1.74. Anterior leg: procoxa = 148 µm; femur = 338 µm; tibia = 334 µm; and tarsomere I = 159 µm; sum of tarsomeres II–V = 258 µm. Median leg: mesocoxa = 232 µm; femur = 666 µm; tibia = 837 µm; and tarsomere I = 369 µm; sum of tarsomeres II–V = 543 µm. Posterior leg: metacoxa = 285 µm; femur = 728 µm; tibia = 1064 µm; and tarsomere I = 491 µm; sum of tarsomeres II–V = 613 µm.

Abdomen: tergites II–VI: presence of randomly distributed setae. Six setae on tergite VIII. Tergite IX without any protuberance. Cerci: 126 μm long. Setae not observed on sternite X. Spermathecae (Fig. 10G and Additional File 12: Supplementary Fig. S12C): common duct short, 10 µm long. Individual ducts of the spermatheca are 145 µm long. Spermathecae are oblong and smooth, 57 µm long and about 33 µm wide. Sessile head.

## Discussion

This study reports the first geographic record of four species of sand flies in Singapore: *Ph. stantoni*, *Se. barraudi* group, *Se. iyengari* group, *Se. whartoni*. In addition, on the basis of integrated morphological and molecular analyses, four new species are described: *Ph. seowpohi* n. sp., *Se. gubleri* n. sp., *Se. leechingae* n. sp., and *Se. retrocalcarae* n. sp.

The majority of sand flies collected are assigned to the new species, *Ph. seowpohi* n. sp. Although the aim of the present study is not to focus on the validity of these species, as their statuses are not universally accepted by all authors, we propose to recognize *Ph. seowpohi* n. sp. as a member of the subgenus *Euphlebotomus* that includes the following species: *Ph. argentipes* s.l.; *Ph. autumnalis*; *Ph. barguesae*; *Ph. caudatus*; *Ph. kiangsuensis*; *Ph. mascomai*; *Ph. mesghalii*; *Ph. nadimi*; *Ph. philippinensis philippinensis*; *Ph. philippinensis gouldi*; *Ph. tumenensis*; and *Ph. yunshengensis* [62–66]. We did not consider *Ph. lengi* originally classified in the subgenus *Larroussius* as a *Euphlebotomus* species [67]. This was previously proposed in 1997 [68], but additional examination of both male and female specimens using morphological and molecular approaches suggested otherwise [66]. Furthermore, *Ph. argentipes* s.l. could be a complex of three species including *Ph. argentipes *s.s., *Ph. annandalei*, and *Ph. glaucus* [69–72].

For *Ph. seowpohi* n. sp. the ratio between the length of its ascoid on the second flagellomere and the length of its second flagellomere (asc/f2 = asc/AIV) was investigated primarily because this character is considered to be the most discriminant by authors working on the variability within the *Ph. argentipes* complex. Female *Ph. seowpohi* n. sp. specimens have an asc/f2 ratio of 0.71, while males have a ratio of 0.61. According to literature, *Ph. argentipes* and *Ph. annandalei* are species with a short f2 ascoid (asc/f2 < 0.5), while *Ph. glaucus* has long ascoids (asc/f2 ranges between 0.50 and 0.60) [71, 72]. Attempts were made to check this character in the type specimens. Unfortunately, access to *Ph. argentipes*- and *Ph. glaucus*-type specimens was not possible. In addition, no type specimen was previously deposited for *Ph. annandalei*.

*Phlebotomus seowpohi* n. sp. possesses two distinct morphological characters. First, both sexes do not exhibit any sensilla on the 3rd flagellomere, which is unusual within the genus *Phlebotomus*. Although earlier descriptions did not mention the absence or presence of such sensilla on the 3rd flagellomere, recent observations of the *Phlebotomus* and *Sergentomyia* genera highlighted the presence of such sensilla in the genus *Phlebotomus* and their absence in the genus *Sergentomyia* [13, 31, 65, 66, 73–75]. This unusual observation suggests that *Ph. seowpohi* n. sp. shares a character associated with the genus *Sergentomyia*. Second, both sexes exhibit one post-alar seta on the mesonotum. The presence of this seta seems unusual but has rarely been documented in taxonomic descriptions published before the experts’ guidelines [54].

To aid future researchers in the identification of *Ph. seowpohi* n. sp. amidst taxonomic challenges in the subgenus *Euphlebotomus*, the differential diagnoses of *Ph. seowpohi* n. sp. from other members of the subgenus are indicated in the following paragraphs.

In females, the pharyngeal armature of *Ph. seowpohi* n. sp. is colorless, whereas it is pigmented in the following *Euphlebotomus* species: *Ph. barguesae*, *Ph. gouldi*, *Ph. kiangsuensis*, *Ph. mascomai*, and *Ph. philippinensis*. The 3rd flagellomere of *Ph. mesghalii* (170–180 µm long) is shorter than those of *Ph. seowpohi* n. sp. (> 220 µm long). The spermathecal ducts of *Ph. seowpohi* n. sp. are smooth, whereas those of *Ph. yunshengensis* are tapeworm-like [76]. The individual spermathecal ducts of *Ph. autumnalis* are longer than the common duct, whereas those in *Ph. seowpohi* n. sp. are shorter than the common duct. The spermatheca of *Ph. tumenensis* is wide and bulbous with faint striations near the duct but without distinct rings. To our knowledge, female specimens of *Ph. caudatus* and *Ph. nadimi* have never been described.

In males, *Ph. autumnalis*, *Ph. caudatus*, *Ph. mesghalii*, and *Ph. nadimi* have a short main paramere lobe. No accessory spine between the parameral sheath and the paramere has been reported in the description of *Ph. mascomai*, and the antennal formula of these species are different [65]. The genitalia of *Ph. tumenensis* do not exhibit any accessory spine between the parameral sheath and the paramere. *Phlebotomus yunshengensis* exhibits a significant tuft of setae on the gonocoxite. The apex of the parameral sheath of male *Ph. barguesae* is blunt-ended, which is not observed in the subgenus *Euphlebotomus*. The ratio between the length of the 3rd flagellomere and the length of the labrum (f3/labrum) differentiates *Ph. seowpohi* n. sp. (about 1.0) from *Ph. philippinensis* and *Ph. gouldi* (> 1.7). The thickness of the middle lobe of the paramere of *Ph. kiangsuensis* should differentiate it from *Ph. seowpohi* n. sp., according to Lewis [48]. However, the taxonomic status of *Ph. kiangsuensis* remains uncertain and warrants further investigation.

*Phlebotomus seowpohi* n. sp. differs from *Ph. argentipes* s.s. by the relative length of their ascoids. The ascoids are long in *Ph. seowpohi* n. sp. and short in *Ph. argentipes*. The differential diagnosis with closely related species of the complex remains impossible until further taxonomic revision of the species complex. The best way to differentiate *Ph. seowpohi* n. sp. from *Ph. argentipes* s.l. is through their highly divergent *cytochrome b* DNA sequences [77–79]. Molecular analyses on the *cytb* and *COI* gene fragments also suggest the validity of *Phlebotomus seowpohi* n. sp. as a new species. Sequences of males and females showed high homology with little variation between sequences. Pairwise intraspecific p-distances were 0.001 and 0.002 for *cytb* and *COI* sequences, respectively (Additional File 14: Supplementary Table S1 and Additional File 16: Supplementary Table S3). This, together with the sequences forming monophyletic clades with high branch support (Fig. 3 and Additional File 2: Supplementary Fig. S2), provides strong evidence that the sequences represent the same species and are distinctly unique from other genetically related species. Moreover, pairwise interspecific p-distances between sequences of *Ph. seowpohi* n. sp. and members of *Euphlebotomus* subgenus had a minimum value of 0.125 and 0.146 for *cytb* and *COI*, respectively (Additional File 14: Supplementary Table S1 and Additional File 16: Supplementary Table S3). This suggests that the genetic sequences of *Ph. seowpohi* n. sp. are at least 12.5% different when compared with sequences of species in the same subgenus. This value exceeds the generally accepted DNA barcoding gap of about 3% used to discriminate between species [77, 80, 81]. Hence, *cytb* and *COI* sequences generated in this study and deposited in Genbank will be valuable for entomological biosurveillance and molecular identification. Molecular DNA barcoding necessitates considerably less technical expertise compared with traditional morphological species identification [81]. Consequently, future researchers will be able to rapidly and accurately identify species by comparing their sequences to those publicly available in online databases.

During the review of specimen inventories, it was determined that *Ph. seowpohi* n. sp. was also recorded in Laos. Specimens from Laos matched the specimens from Singapore in both morphological and molecular data (Fig. 3 and Additional File 2: Supplementary Fig. S2). The distribution range of this new species thus requires further evaluation. *Phlebotomus seowpohi* n. sp. could potentially be widely distributed across SEA. Considering the description of *Ph. seowpohi* n. sp. in both countries, previous records of *Ph. argentipes s. l.* in SEA therefore remain doubtful. We propose a systematic taxonomic study of all the specimens caught in this region to ascertain the distribution of these species and detect possible *Ph. argentipes* misidentifications in previous entomological surveys.

In Bangladesh, India, Nepal, and Bhutan, *Ph. argentipes* is an important vector of *Leishmania donovani* and is recognized as the primary vector responsible for transmitting human visceral leishmaniasis in the South Asian region [82, 83]. The discovery of *Ph. seowpohi* n. sp. has significant public health importance, as evidence from our integrative taxonomic analyses indicates a close phylogenetic relationship with *Ph. argentipes*. Comparative analyses between *Ph. seowpohi* n. sp. and its *Leishmania*-transmitting relative may elucidate the genetic, ecological, and physiological determinants of vector competence. Accordingly, further research on *Ph. seowpohi* n. sp. is essential to assess its potential role as a vector of leishmaniasis.

Besides *Ph. seowpohi* n. sp., *Ph. stantoni* was the only other *Phlebotomus* species collected in Singapore (Additional File 5: Supplementary Fig. S5 and Additional File 6: Supplementary Fig. S6). This species is known to be widely distributed across SEA [24, 25, 30, 84]. On the basis of the ML tree generated from this study, the specimens from Singapore clearly clustered together with other *Ph. stantoni* collected from the region.

Unlike the genus *Phlebotomus*, which is well characterized, the genus *Sergentomyia* is considered a catch-all group by many phlebotomine sand fly taxonomists [8, 85]. Its systematics remain debatable and require revision. For this reason, we do not propose the inclusion of the new species we described here into any subgenus. Useful taxonomic notes on *Sergentomyia* species collected in Singapore, including the newly described species, can be found in the following paragraphs.

**Fig. 11:**
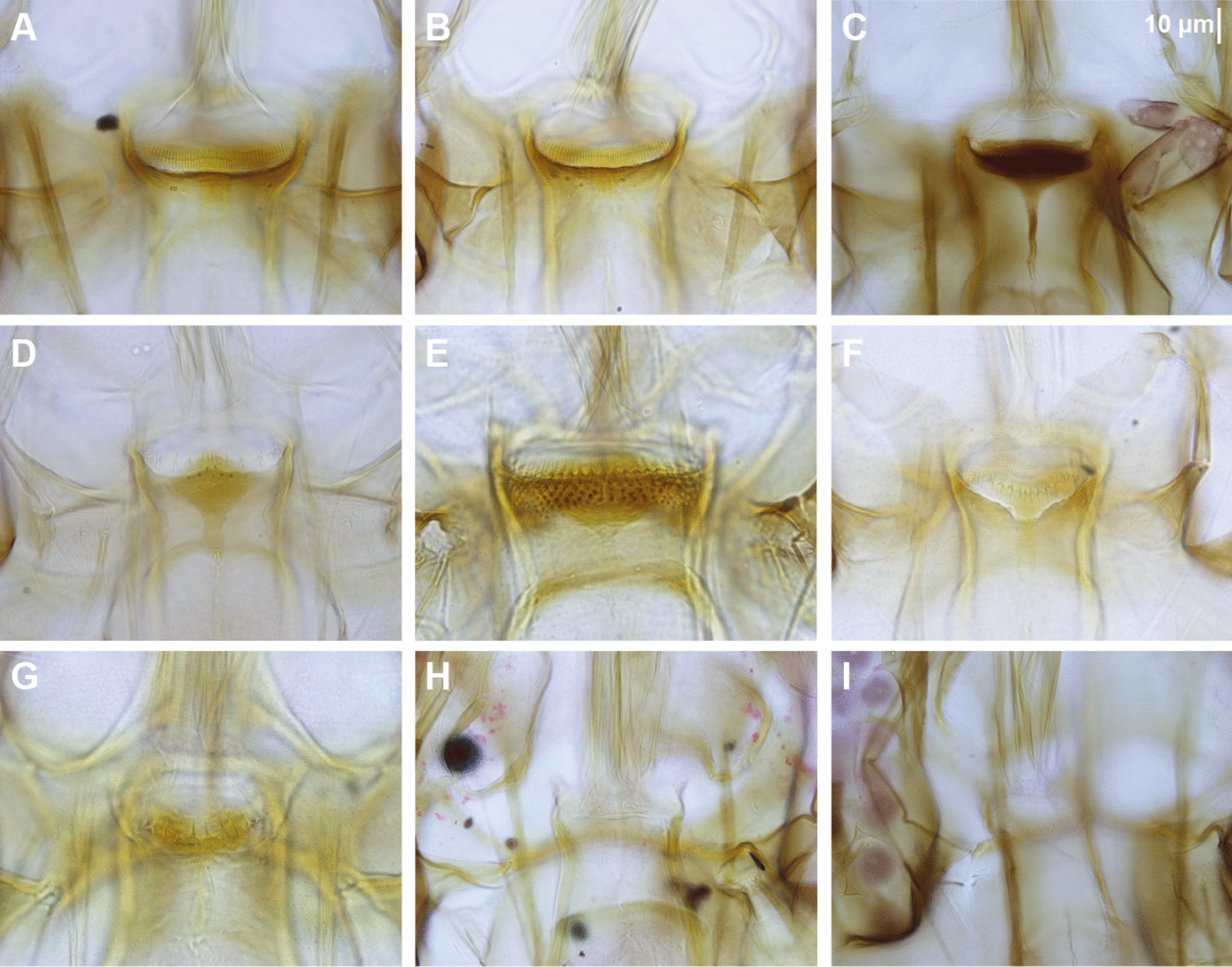
Cibarium of female sand flies collected in Singapore. All photos are at the same scale: the 10 µm bar can be applied to each photo. **A** *Sergentomyia barraudi* group NEA0501.1, **B** *Se. barraudi* group NEA0009.2, **C** *Se. gubleri* n. sp. NEA1119.1.BF1, **D** *Se. iyengari* group NEA0283, **E** *Se. retrocalcarae* n. sp. NEA0120.1, **F** *Se. leechingae* n. sp. NEA0118.1, **G** *Se. whartoni* NEA0069.1, **H** *Phlebotomus stantoni* NEA0569, **I** *Ph. seowpohi* n. sp. NEA0149.3

*Sergentomyia gubleri* n. sp. exhibits many cibarial teeth similar to *Se. rudnicki* and *Se. brevicaulis*, which are members of the *Se. barraudi* group. However, this new species is molecularly distant from all other members of the group. It can be distinguished by the length of the first flagellomere and by the number of teeth constituting the comb-like cibarial armature (Table 2 and Fig. 11).

**Table 2:** Differential characters of *Sergentomyia gubleri* n. sp. compared against closely related species *Se. rudnicki* and *Se. brevicaulis*

Characters	*Se. gubleri* n. sp.	*Se. rudnicki*	*Se. brevicaulis*
Flagellomere 1	250 µm	290 (260–330) µm	380 µm
Posterior teeth on cibarium	68	90	50
Anterior teeth on cibarium	One row of 15	Two rows of 22	One row
Sclerotized area within the cibarium	Not reaching the buccal walls	Not reaching the buccal walls	Reaching the buccal walls
Pharynx	Very long teeth	Very long teeth	Long teeth
Newstead’s sensilla on palpal segment 3 (p3)	Few on basal half of p3	Few on basal half of p3	Few on basal quarter of p3

*Sergentomyia leechingae* n. sp. exhibits a cibarial notch similar to *Se. babu*, *Se. insularis*, *Se. baghdadis*, and *Se. shortii*, but it is less developed (Fig. 11). These species can be distinguished by the shape of their cibarial notch and the number of teeth on the cibarial armature as indicated in Table 3.

**Table 3:** Differential characters of female *Sergentomyia leechingae* n. sp. and related species

Characters	*Se. leechingae* n. sp	*Se. babu*	*Se. insularis*	*Se. baghdadis*	*Se. shortii*
Posterior teeth on cibarium	14	24–34	45–50	16–20	10–14 in a straight line
Cibarial notch	Shallow	Deep	Deep	Deep	Shallow

*Sergentomyia retrocalcarae* n. sp. exhibit spurs on the ascoids that are oriented backward on the flagellomeres. It also has a cibarium that has many anterior teeth. However, the number of cibarial teeth in this species is fewer than those of *Se. gombaki* and *Se. arboris*, both of which also exhibit spurs on the ascoids. The differential diagnosis with closely related species is provided in Table 4.

**Table 4:** Differential characters of female *Sergentomyia retrocalcarae* n. sp. and related species

Characters	*Se. retrocalcarae* n. sp.	*Se. gemmea*	*Se. malayae*	*Se raynali*
Ascoid	Short spur	Short spur	Very long spur	Short spur
Anterior teeth on cibarium	Three rows, first row not very large	Three rows, first row very large	Three rows, first row not very large	Two rows, first row not very large; few anterior teeth

The *Sergentomyia barraudi* group is undeniably a complex species that should be explored further by investigating both morphological and molecular characters of individuals collected from various populations across its distribution area (Additional File 7: Supplementary Fig. S7). Morphological variability can be observed in the varying number and distribution of cibarial teeth (Fig. 11), while the molecular variability is demonstrated by genetic differences between mitochondrial barcodes (Fig. 4, Additional File 2: Supplementary Fig. S2, Additional File 15: Supplementary Table S2, and Additional File 16: Supplementary Table S4). The specimens from Singapore have more posterior teeth (56–60) than those in the original description (40) [86]. Hence, we have identified the specimens collected in the current study as belonging to the *Se. barraudi* group, as a more precise identification could be erroneous.

Many records of *Se. iyengari* have been made in Southeastern Asia. However, this species was described from the southernmost part of India, far from the SEA region [87]. In our opinion, all records of *Se. iyengari* in SEA should in fact refer to either *Se. khawi* or *Se. hivernus*. The species *Se. tambori* Lewis and Jeffery, 1978 described from Malaysia could also be included in the *iyengari* group. As with the *barraudi* group discussed above, the *iyengari* group requires revision, incorporating various populations from India and Southeast Asia. To avoid further confusion and taxonomic errors, we prefer to identify specimens collected from Singapore as belonging to the *Se. iyengari* group (Additional File 9: Supplementary Fig. S9). A photograph of the cibarial armature of the specimen collected in this study is provided in Fig. 11.

The specimen labeled NEA0069.1 resembles *Se. whartoni* described from continental Malaysia, about 200 km north of Singapore [88]. Its morphological characters match the original description of *Se. whartoni*. Although we observed that the *COI* barcode of NEA0069.1 is similar to specimens identified as *Se. perturbans* from Thailand (mean interspecific p-distance 2.2%) (Additional File 15: Supplementary Table S2), we took into account the complex history of *Se. perturbans* [89–91] and Lewis’s consideration of *Se. whartoni* as a junior synonym of *Se. perturbans* [87]. We therefore considered the specimen from Singapore as *Se. whartoni* (Additional File 13: Supplementary Fig. S13), given that the identity of *Se. perturbans* remains doubtful. A photograph of specimen NEA0069.1 can be found in Fig. 11.

## Conclusions

To the best of our knowledge, this is the first report on the presence of phlebotomine sand flies in Singapore. This study describes four new species of sand flies and reports new geographic records of one *Phlebotomus* sp. and three *Sergentomyia* sp. The newly identified species are *Ph. seowpohi* n. sp., *Se. gubleri* n. sp., *Se. leechingae* n. sp., and *Se. retrocalcarae* n. sp. In addition, new distributional records have been documented for previously known species, namely: *Ph. stantoni*, *Se. barraudi* group, *Se. iyengari* group, and *Se. whartoni*. Sand flies were found across secondary forests, public parks, peri-urban vegetated areas, open fields, and coastal areas (Fig. 2 and Additional File 1: Supplementary Fig. S1). These locations represent only the current sampling sites and do not necessarily represent all areas where sand flies occur in Singapore.

Although Singapore is not endemic for human leishmaniasis, its vulnerability to the disease is heightened by its role as a major travel hub, substantial reliance on migrant workers from endemic regions, and increased travel to leishmaniasis-endemic areas. Documented cases of imported leishmaniasis among migrant workers and returning travelers highlight the risk of introducing *Leishmania* and other pathogens into the country [92–94]. In 2020, antibodies to *Leishmania infantum*—which cause zoonotic leishmaniasis, were detected in a local free-roaming dog [95]. Dogs are known to be the main reservoir hosts of the disease [96–99]. This finding, coupled with the risk of human leishmaniasis being introduced into Singapore and changes driven by urbanization, land use change, and climate change [42, 100–105], underscore the importance of understanding local sand fly biology, ecology, diversity, distribution, and disease transmission potential. Strengthening biosurveillance efforts, by expanding surveillance sites, and conducting continuous monitoring of sand fly populations, will help fill existing knowledge gaps and provide deeper insights into local sand fly fauna.

## Supplementary Information

Additional file 1: Fig. S1 Twenty-nine sampling sites across Singapore where entomological surveillance was performed in this study. Each colored dot represents a site and its corresponding habitat type.

Additional file 2: Fig. S2 Maximum-likelihood A *Phlebotomus*, and B *Sergentomyia* phylogenetic tree inferred from aligned consensus *cytochrome c oxidase subunit I* (*COI*) sequences using the Hasegawa-Kishino-Yano 85 model. Sequences highlighted in red are generated from specimens collected in Singapore. Sequence in yellow is from Luangphabang, Laos, as part of IP Laos collection program. It will be further detailed in the discussion section. The trees have been rooted on reference *Sergentomyia barraudi* and *Phlebotomus stantoni* sequences which are selected as outgroups, respectively. Nodes with bootstrap value less than 70% are not shown.

Additional file 3: Fig. S3 *Phlebotomus seowpohi* n. sp. female. A Pharynx (voucher NEA0149.3), B spermathecae in phase contrast (voucher NEA0149.4). Bars=20 µm.

Additional file 4: Fig. S4 *Phlebotomus seowpohi* n. sp. male. A Pharynx (voucher NEA0389.3), B cibarium in phase contrast (voucher NEA0149.7), C genitalia showing paramere and parameral sheath (voucher NEA0076.1), D accessory spine (voucher NEA0149.7). Bars=20 µm except for photograph C (bar=100µm).

Additional file 5: Fig. S5 *Phlebotomus stantoni* female (voucher NEA0140). A Pharynx, B spermathecae. Bars=20 µm.

Additional file 6: Fig. S6 *Phlebotomus stantoni* male (voucher NEA0079). A Pharynx, B genitalia, C cibarium. Bars=20 µm.

Additional file 7: Fig. S7 *Sergentomyia barraudi* group female. A Pharynx (voucher NEA0501.1), B spermathecae in phase contrast (voucher NEA0009.2). Bars=20 µm.

Additional file 8: Fig. S8 *Sergentomyia gubleri *n. sp. female (voucher NEA1119.7.BF1). A Pigmented mouthpart, B pharynx, C eggs, D common duct of the spermathecae, E body of the spermathecae. Bars=20 µm.

Additional file 9: Fig. S9 *Sergentomyia iyengari* group female. A Pharynx (voucher NEA0697.0.1), B spermathecae in phase contrast (voucher NEA0141). Bars=20 µm.

Additional file 10: Fig. S10 *Sergentomyia leechingae* n. sp. female (voucher NEA0118.1) in phase contrast. A Pharynx, B spermathecae. Bars=20 µm.

Additional file 11: Fig. S11 *Sergentomyia leechingae* n. sp. male (voucher NEA0118.4). A Cibarium in phase contrast, B genitalia. Bars=20 µm.

Additional file 12: Fig. S12 *Sergentomyia retrocalcarae* n. sp. female (voucher NEA0120.1) in phase contrast. A 2^nd^ flagellomere exhibiting the spur on the ascoid indicated by the arrow, B pharynx, C spermathecae. Bars=20 µm.

Additional file 13: Fig. S13 *Sergentomyia whartoni* female (voucher NEA0069.1). A Spermathecae in phase contrast, B pharynx, D pigmented matafurca. Bars=20 µm.

Additional file 14: Table S1 Mean interspecific genetic distances for *cytb* sequence pairs between five *Phlebotomus (Euphlebotomus)* species and *Ph. (Anaphlebotomus) stantoni*. Numbers (1-8) in the top row correspond to the species listed. Calculations were based on the p-distance model. Diagonal bold values indicate intraspecific mean distances. Names with a hashtag refer to specimens collected in Singapore (SG).

Additional file 15: Table S2 Mean interspecific genetic distances for *cytb* sequence pairs between *Sergentomyia* species and *Grassomyia* species. Numbers (1-17) in the top row correspond to the species listed. Calculations were based on the p-distance model. Diagonal bold values indicate intraspecific mean distances. Names with a hashtag refer to specimens collected in Singapore (SG). NA denotes cases in which it was not possible to estimate genetic distances due to single DNA barcode.

Additional file 16: Table S3 Mean interspecific genetic distances for *COI* sequence pairs between four *Phlebotomus* (*Euphlebotomus*) species and *Ph.* (*Anaphlebotomus*) *stantoni*. Numbers (1-7) in the top row correspond to the species listed. Calculations were based on the p-distance model. Diagonal bold values indicate intraspecific mean distances. Names with a hashtag refer to specimens collected in Singapore (SG). NA denotes cases in which it was not possible to estimate genetic distances due to single DNA barcode.

Additional file 17: Table S4 Mean interspecific genetic distances for *COI* sequence pairs between *Sergentomyia* species. Numbers (1-17) in the top row correspond to the species listed. Calculations were based on the p-distance model. Diagonal bold values indicate intraspecific mean distances. Names with a hashtag refer to specimens collected in Singapore (SG). NA denotes cases in which it was not possible to estimate genetic distances due to single DNA barcode.

Additional file 18: Table S5 Morphometric measurements (in µm) of *Phlebotomus seowpohi* n. sp. specimens. Values are presented as mean (minimum-maximum).

## Data Availability

All data generated or analyzed during this study are included in this published article. The newly generated sequences were deposited in the GenBank database under the following accession nos. PQ468888–PQ468953 (*COI*), PQ894477–PQ894506 (*cytb*), and PQ151942–PQ151977 (*cytb*).

## Abbreviations

AIC: Aikaike information criterion
*COI*: *Cytochrome c oxidase subunit I*
*cytb*: *Cytochrome b*
ML: Maximum likelihood
NC: Night Catcher
NEA: National Environment Agency
NTD: Neglected tropical disease
PCR: Polymerase chain reaction
SEA: Southeast Asia
