# Supplementary material for: Hidden in plain sight: discovery of sand flies in Singapore and description of four species new to science
Source: Parasit Vectors. 2025 Oct 9;18:402. doi: 10.1186/s13071-025-07021-5 (PMC12512794; doi:10.1186/s13071-025-07021-5)
Supplement: Supplementary file 1 — Additional file 1: Fig. S1 Twenty-nine sampling sites across Singapore where entomological surveillance was performed in this study. Each colored dot represents a site and its corresponding habitat type. [file 13071_2025_7021_MOESM1_ESM.docx]

**Additional file 1: Fig. S1** Twenty-nine sampling sites across Singapore where entomological surveillance was performed in this study. Each colored dot represents a site and its corresponding habitat type.

**
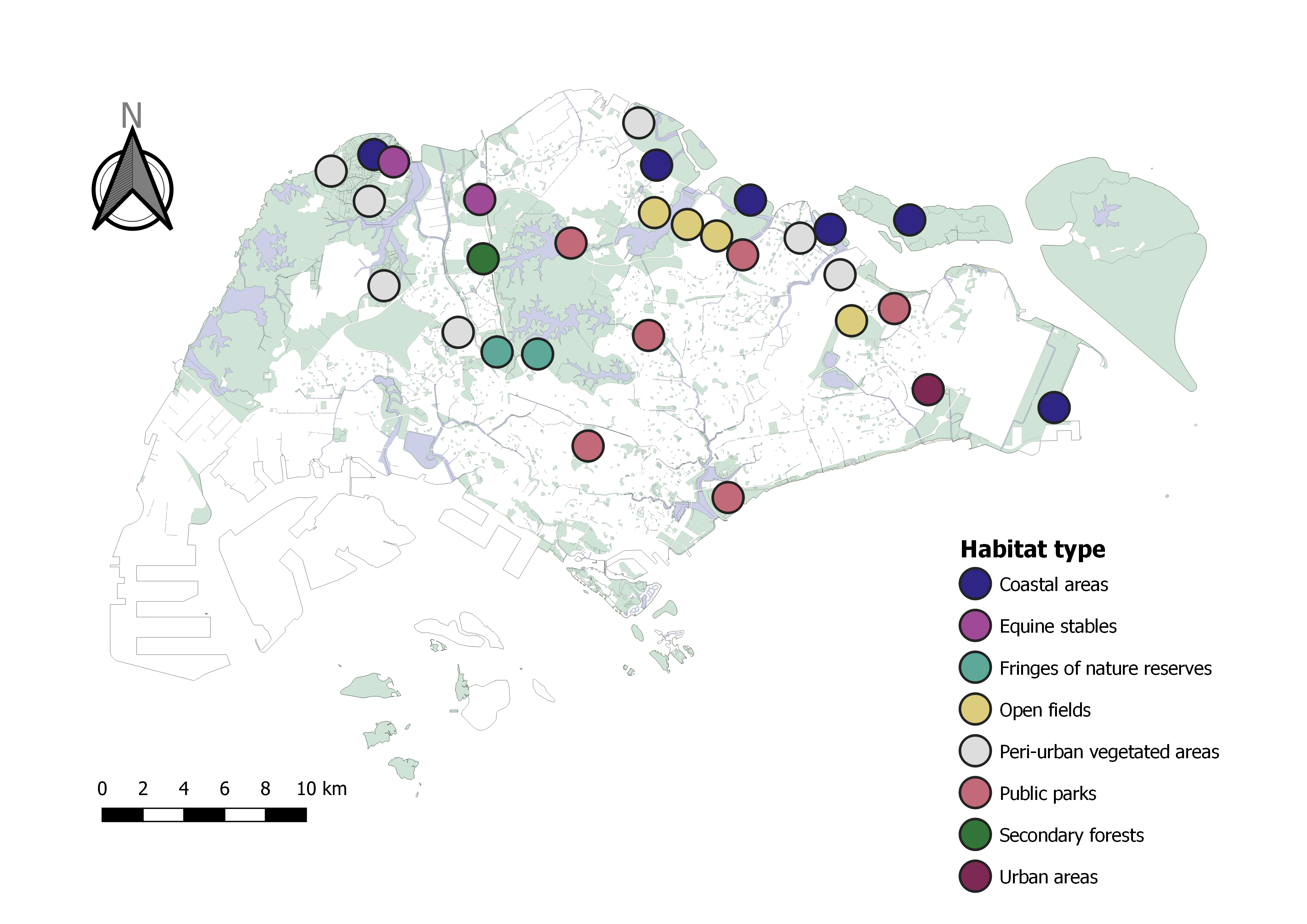
**
