# Supplementary material for: Hidden in plain sight: discovery of sand flies in Singapore and description of four species new to science
Source: Parasit Vectors. 2025 Oct 9;18:402. doi: 10.1186/s13071-025-07021-5 (PMC12512794; doi:10.1186/s13071-025-07021-5)

**Additional file 2: Fig. S2** Maximum-likelihood **A** *Phlebotomus* and, **B** *Sergentomyia* phylogenetic tree inferred from aligned consensus *cytochrome c oxidase subunit I* (*COI*) sequences using the Hasegawa-Kishino-Yano 85 model. Sequences highlighted in red are generated from specimens collected in Singapore. Sequence in yellow is from Luangphabang, Laos, as part of IP Laos collection program. It will be further detailed in the discussion section. The trees have been rooted on reference *Sergentomyia barraudi* and *Phlebotomus stantoni* sequences which are selected as outgroups, respectively. Nodes with bootstrap value less than 70% are not shown.


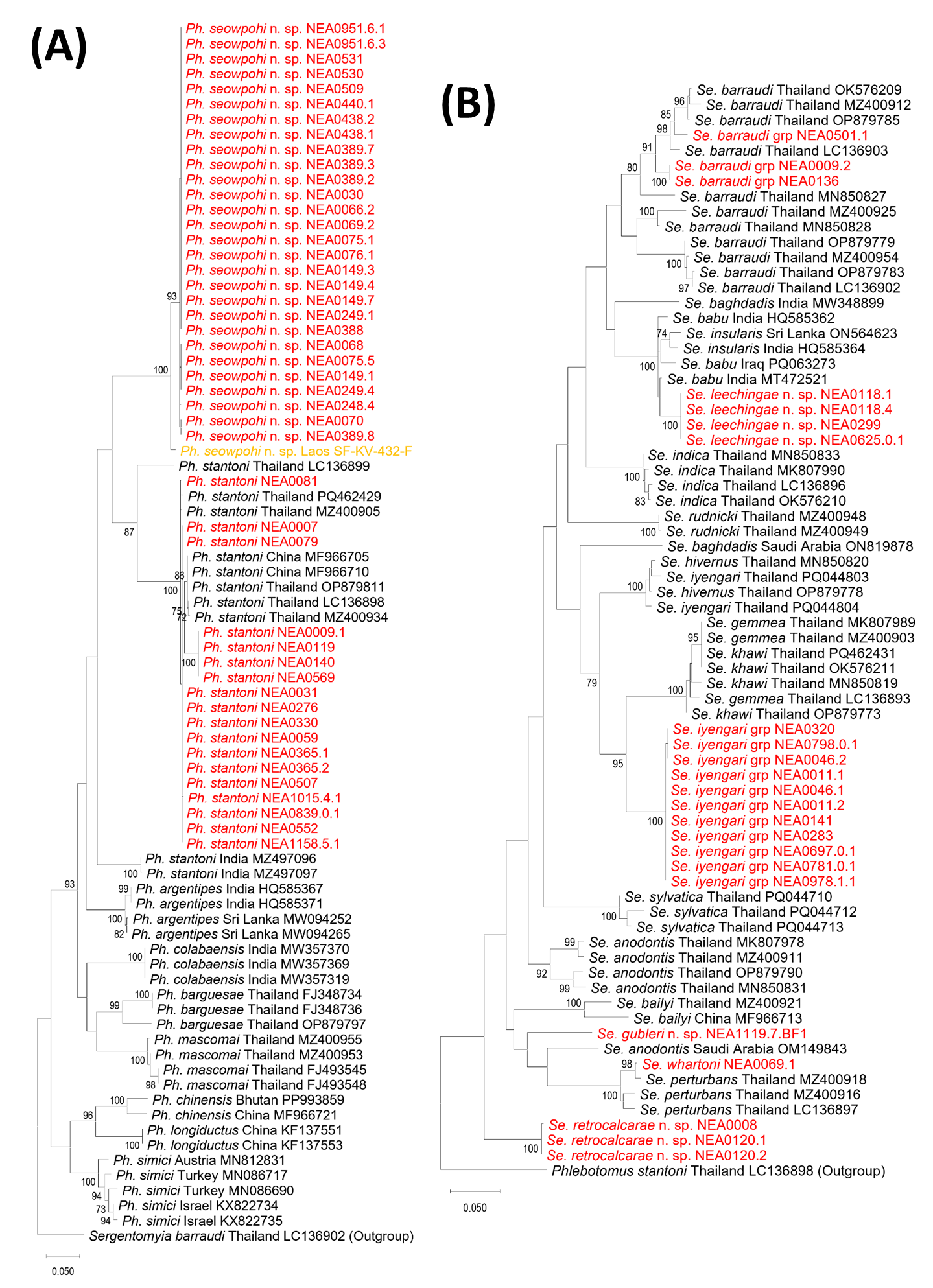

Supplement: Supplementary file 2 — Additional file 2: Fig. S2 Maximum-likelihood A Phlebotomus, and B Sergentomyia phylogenetic tree inferred from aligned consensus cytochrome c oxidase subunit I (COI) sequences using the Hasegawa-Kishino-Yano 85 model. Sequences highlighted in red are generated from specimens collected in Singapore. Sequence in yellow is from Luangphabang, Laos, as part of IP Laos collection program. It will be further detailed in the discussion section. The trees have been rooted on reference Sergentomyia barraudi and Phlebotomus stantoni sequences which are selected as outgroups, respectively. Nodes with bootstrap value less than 70% are not shown. [file 13071_2025_7021_MOESM2_ESM.docx]
