# Supplementary figures and images for: Hidden in plain sight: discovery of sand flies in Singapore and description of four species new to science
Source: Parasit Vectors. 2025 Oct 9;18:402. doi: 10.1186/s13071-025-07021-5 (PMC12512794; doi:10.1186/s13071-025-07021-5)

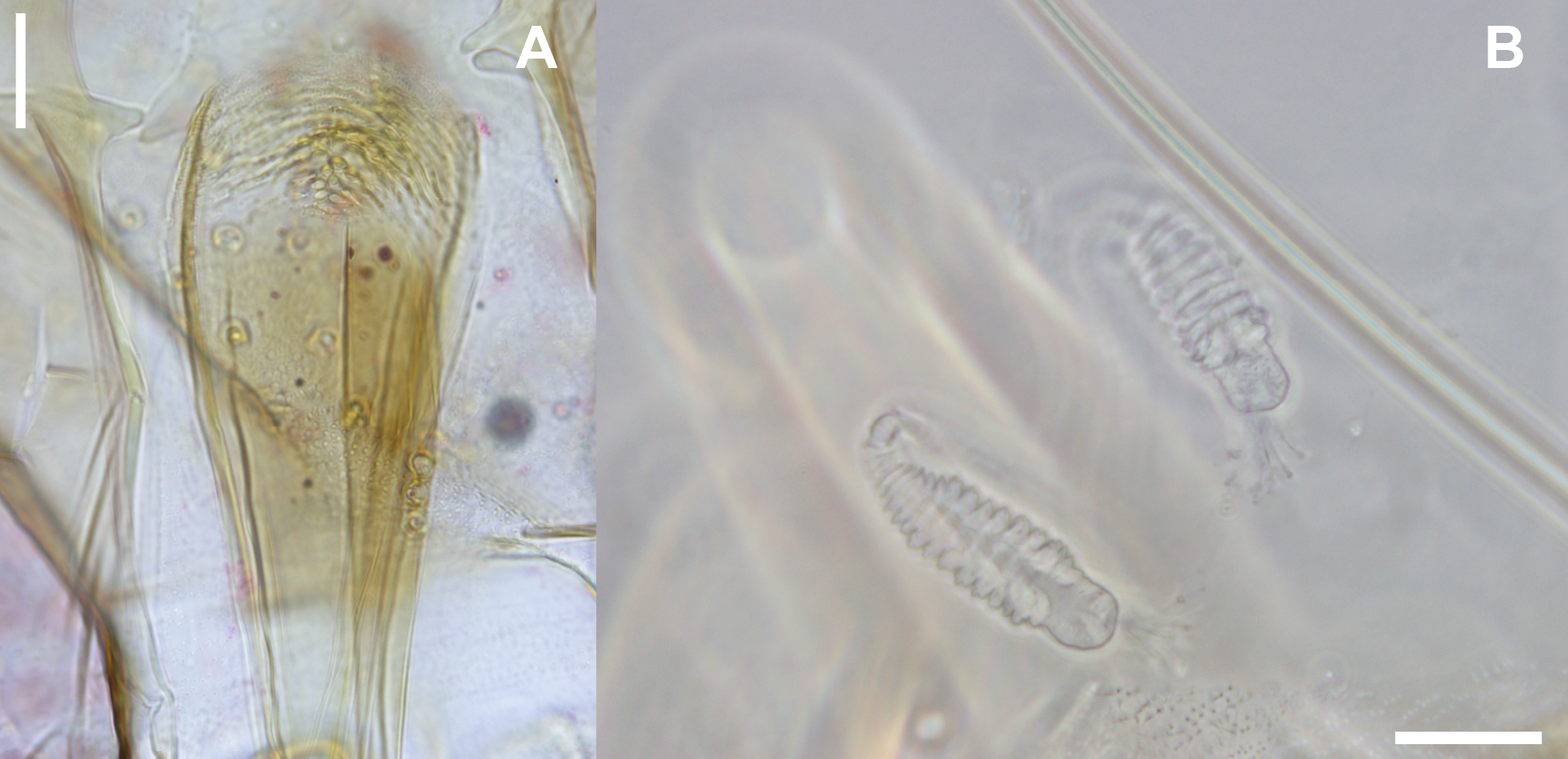

Supplement: Supplementary file 3 — Additional file 3: Fig. S3 Phlebotomus seowpohi n. sp. female. A Pharynx (voucher NEA0149.3), B spermathecae in phase contrast (voucher NEA0149.4). Bars=20 µm. [file 13071_2025_7021_MOESM3_ESM.png]

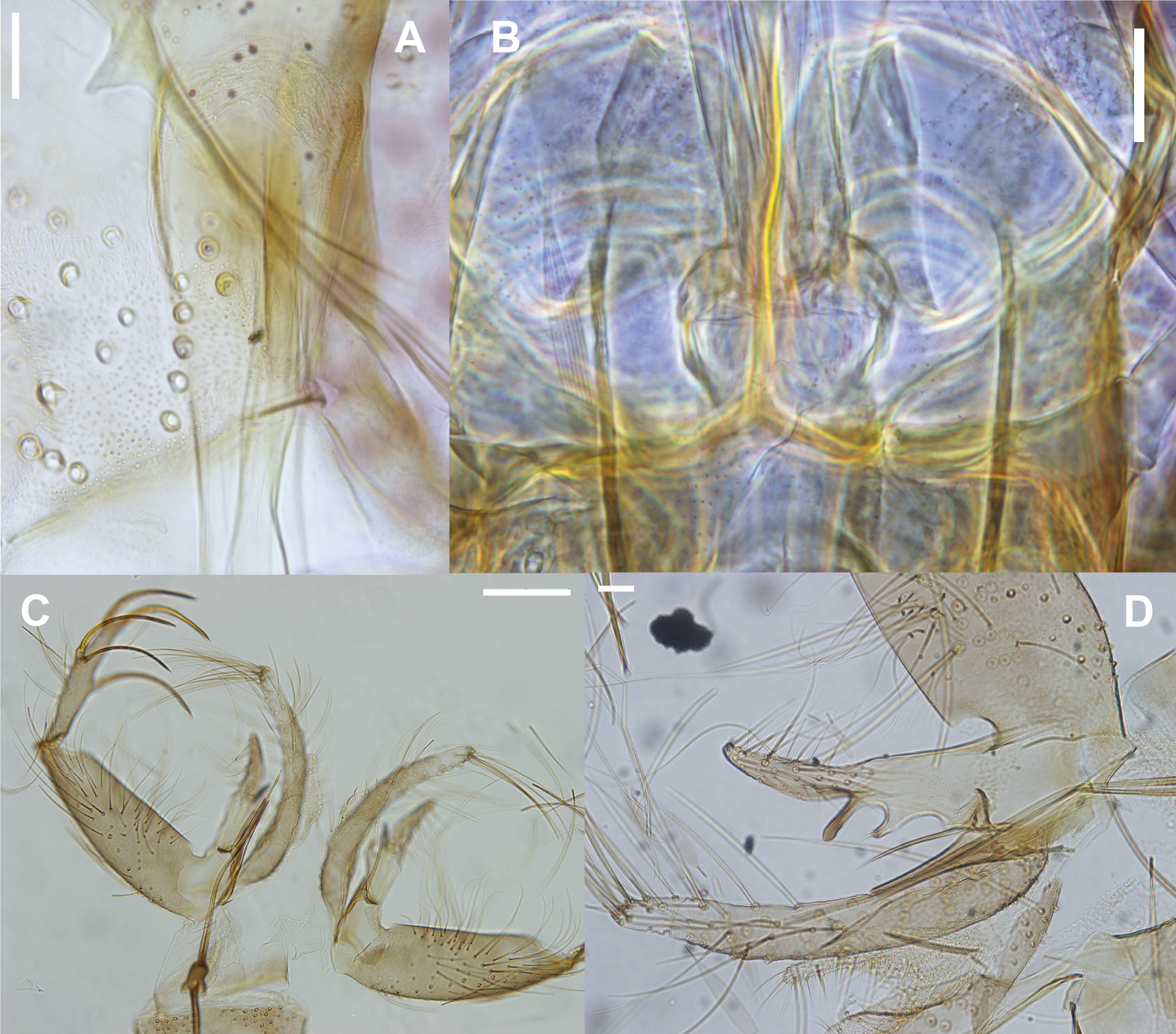

Supplement: Supplementary file 4 — Additional file 4: Fig. S4 Phlebotomus seowpohi n. sp. male. A Pharynx (voucher NEA0389.3), B cibarium in phase contrast (voucher NEA0149.7), C genitalia showing paramere and parameral sheath (voucher NEA0076.1), D accessory spine (voucher NEA0149.7). Bars=20 µm except for photograph C (bar=100µm). [file 13071_2025_7021_MOESM4_ESM.png]

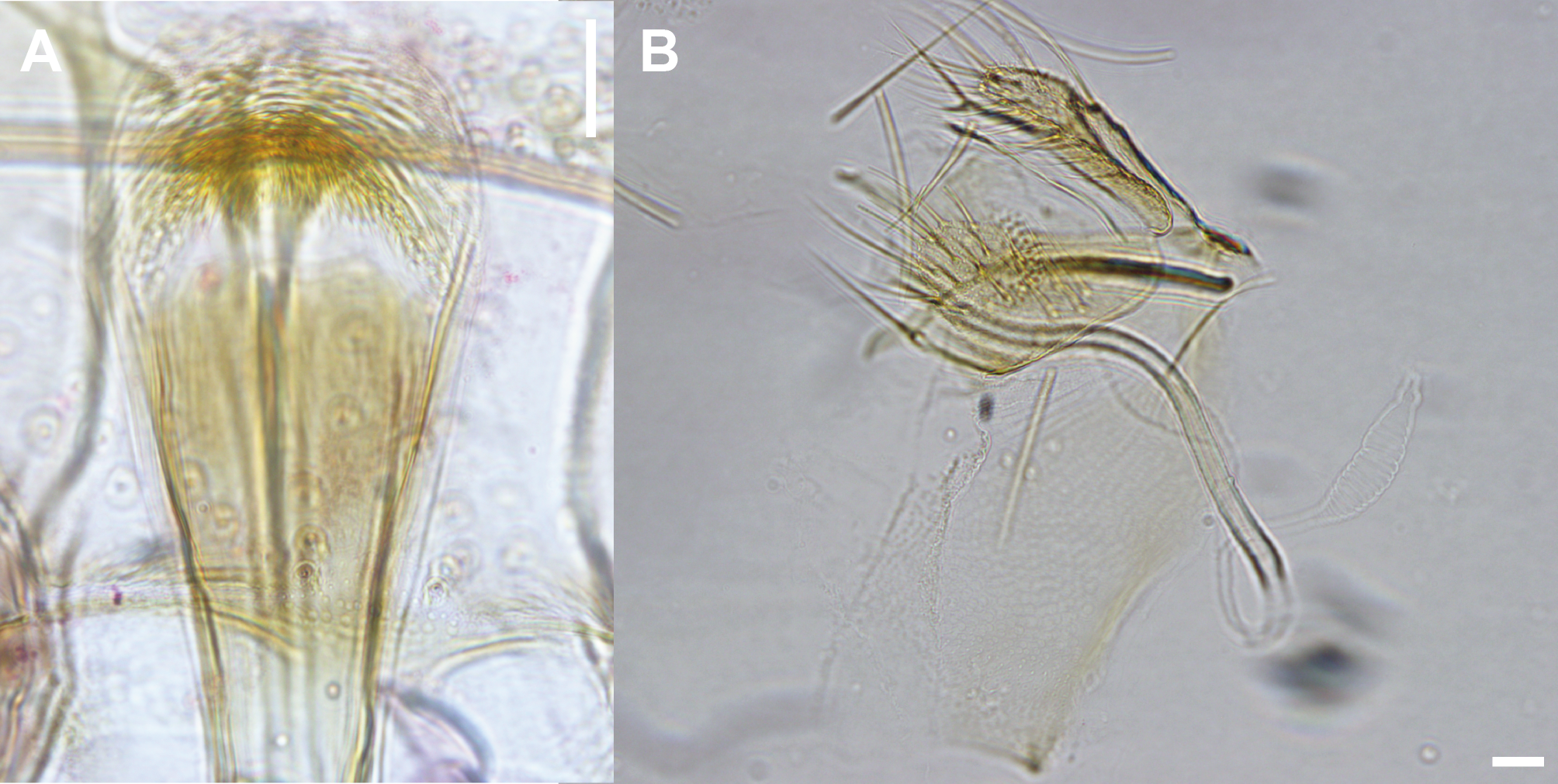

Supplement: Supplementary file 5 — Additional file 5: Fig. S5 Phlebotomus stantoni female (voucher NEA0140). A Pharynx, B spermathecae. Bars=20 µm. [file 13071_2025_7021_MOESM5_ESM.png]

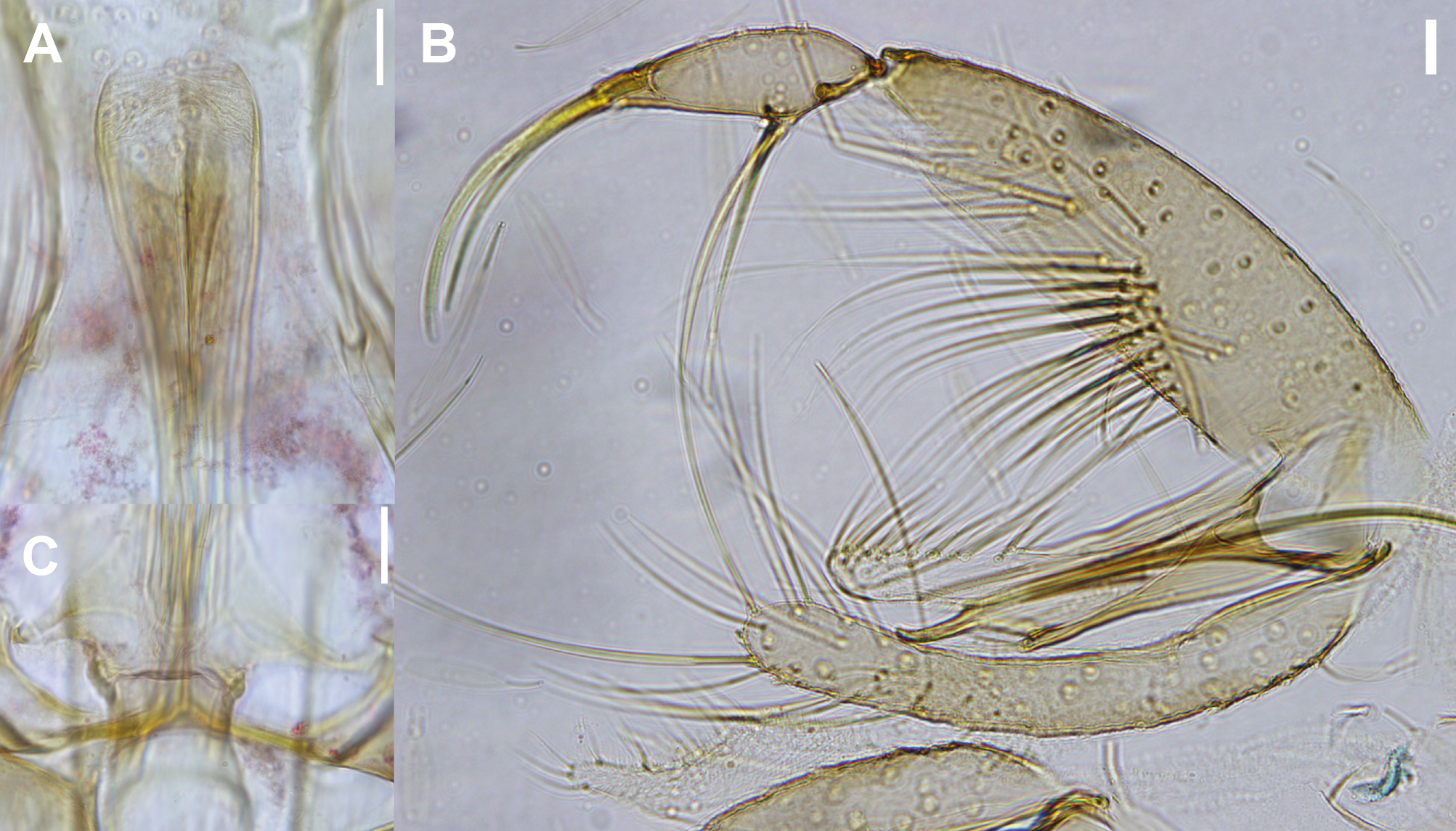

Supplement: Supplementary file 6 — Additional file 6: Fig. S6 Phlebotomus stantoni male (voucher NEA0079). A Pharynx, B genitalia, C cibarium. Bars=20 µm. [file 13071_2025_7021_MOESM6_ESM.png]

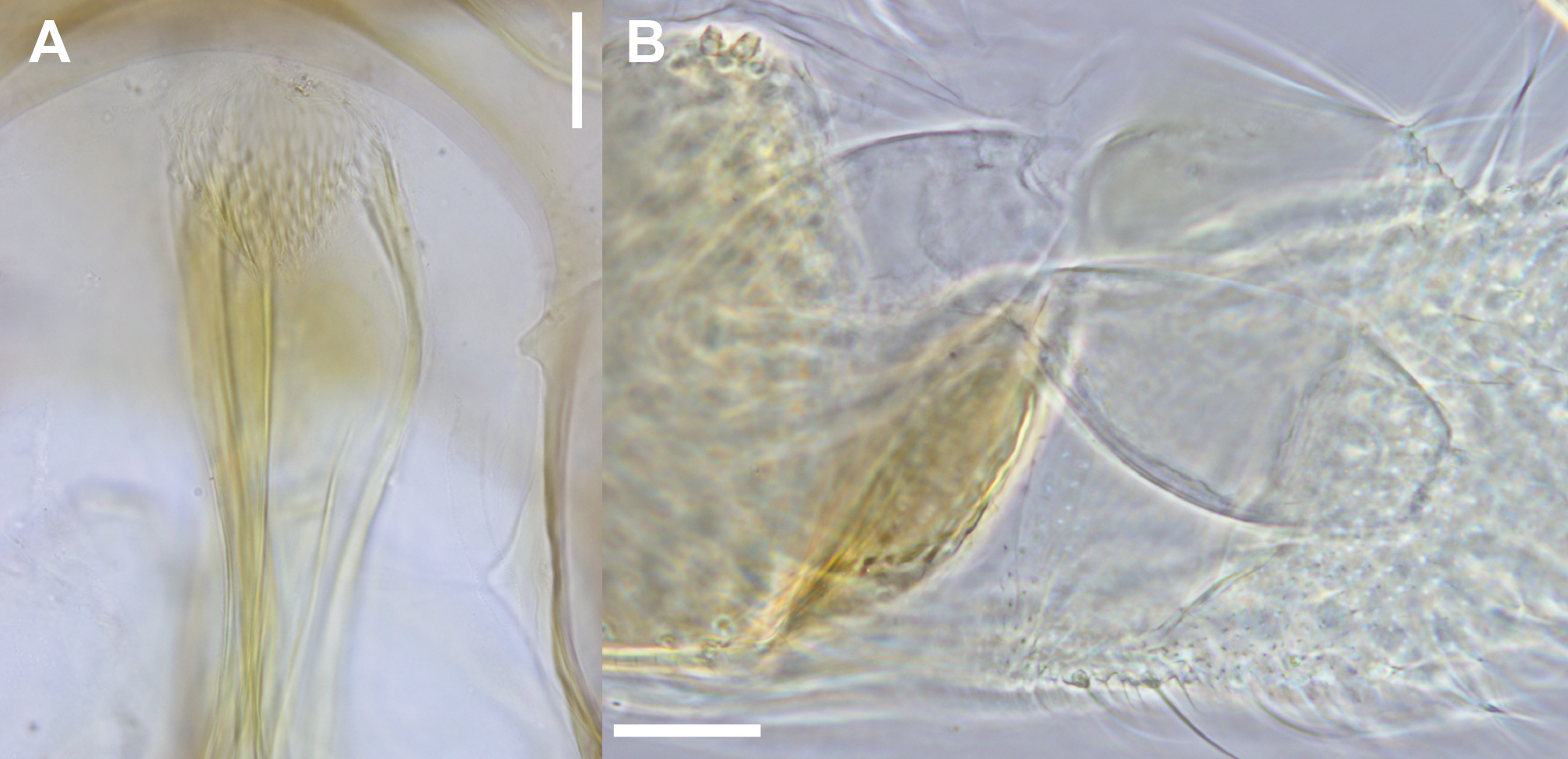

Supplement: Supplementary file 7 — Additional file 7: Fig. S7 Sergentomyia barraudi group female. A Pharynx (voucher NEA0501.1), B spermathecae in phase contrast (voucher NEA0009.2). Bars=20 µm. [file 13071_2025_7021_MOESM7_ESM.png]

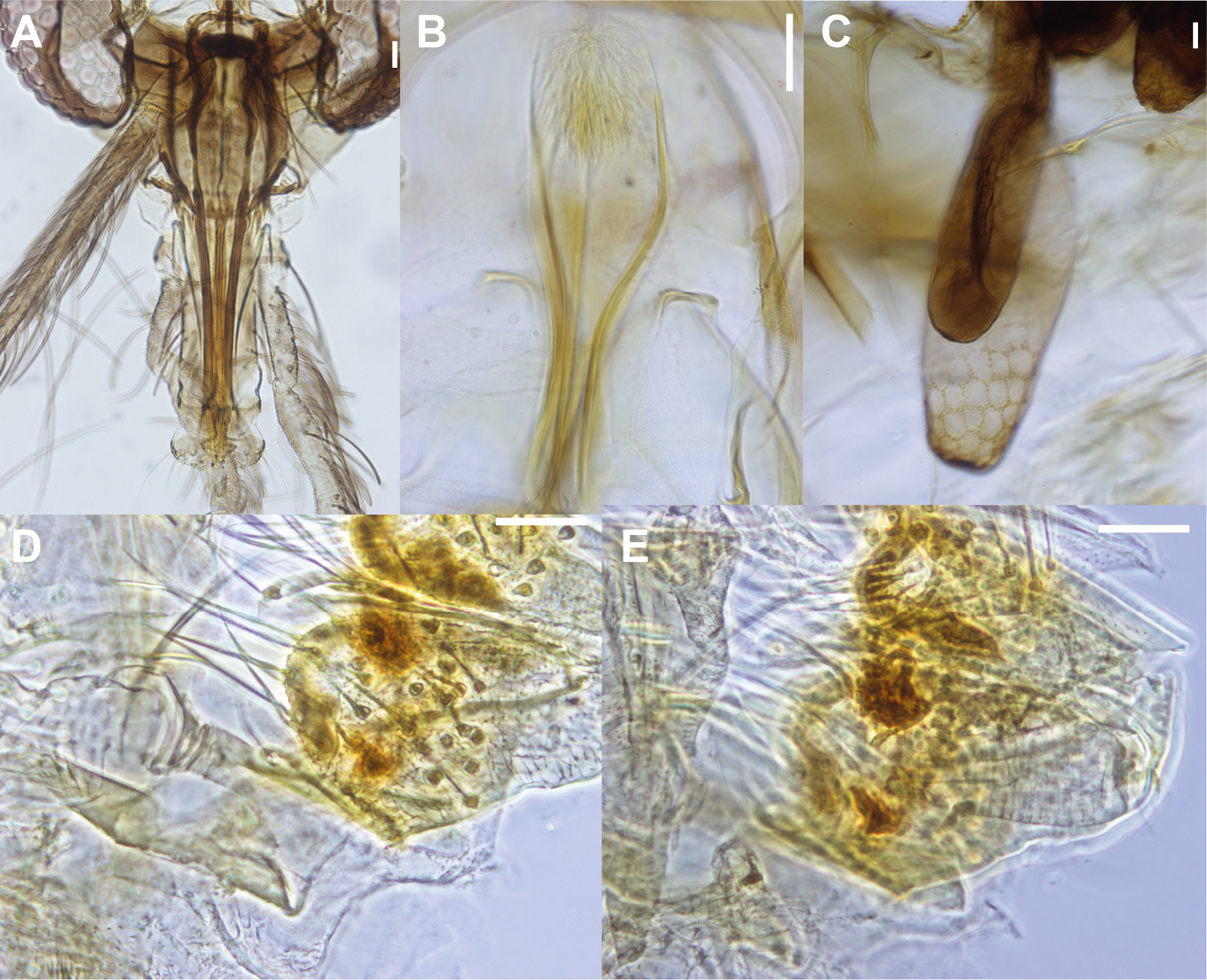

Supplement: Supplementary file 8 — Additional file 8: Fig. S8 Sergentomyia gubleri n. sp. female (voucher NEA1119.7.BF1). A Pigmented mouthpart, B pharynx, C eggs, D common duct of the spermathecae, E body of the spermathecae. Bars=20 µm. [file 13071_2025_7021_MOESM8_ESM.png]

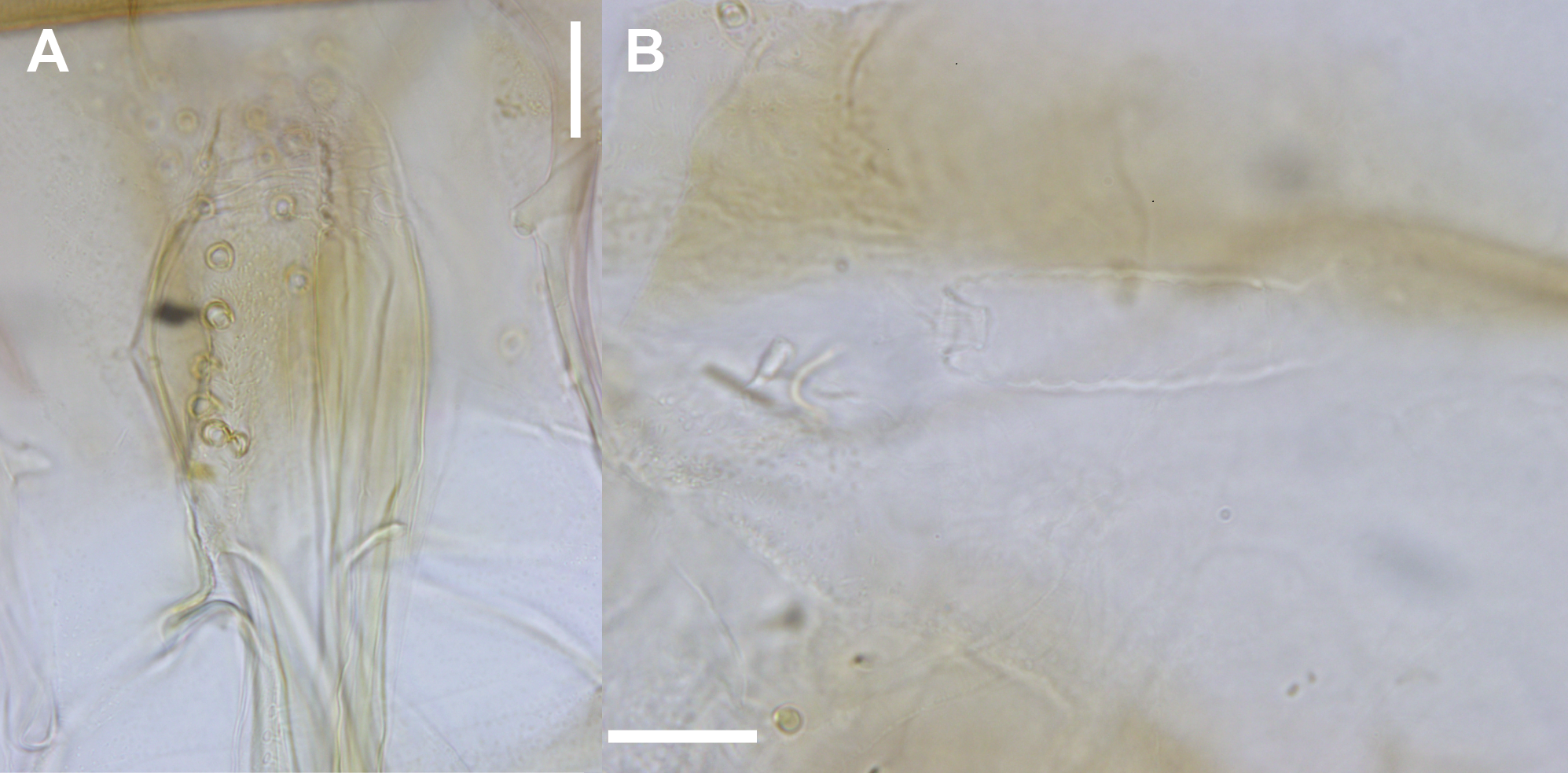

Supplement: Supplementary file 9 — Additional file 9: Fig. S9 Sergentomyia iyengari group female. A Pharynx (voucher NEA0697.0.1), B spermathecae in phase contrast (voucher NEA0141). Bars=20 µm. [file 13071_2025_7021_MOESM9_ESM.png]

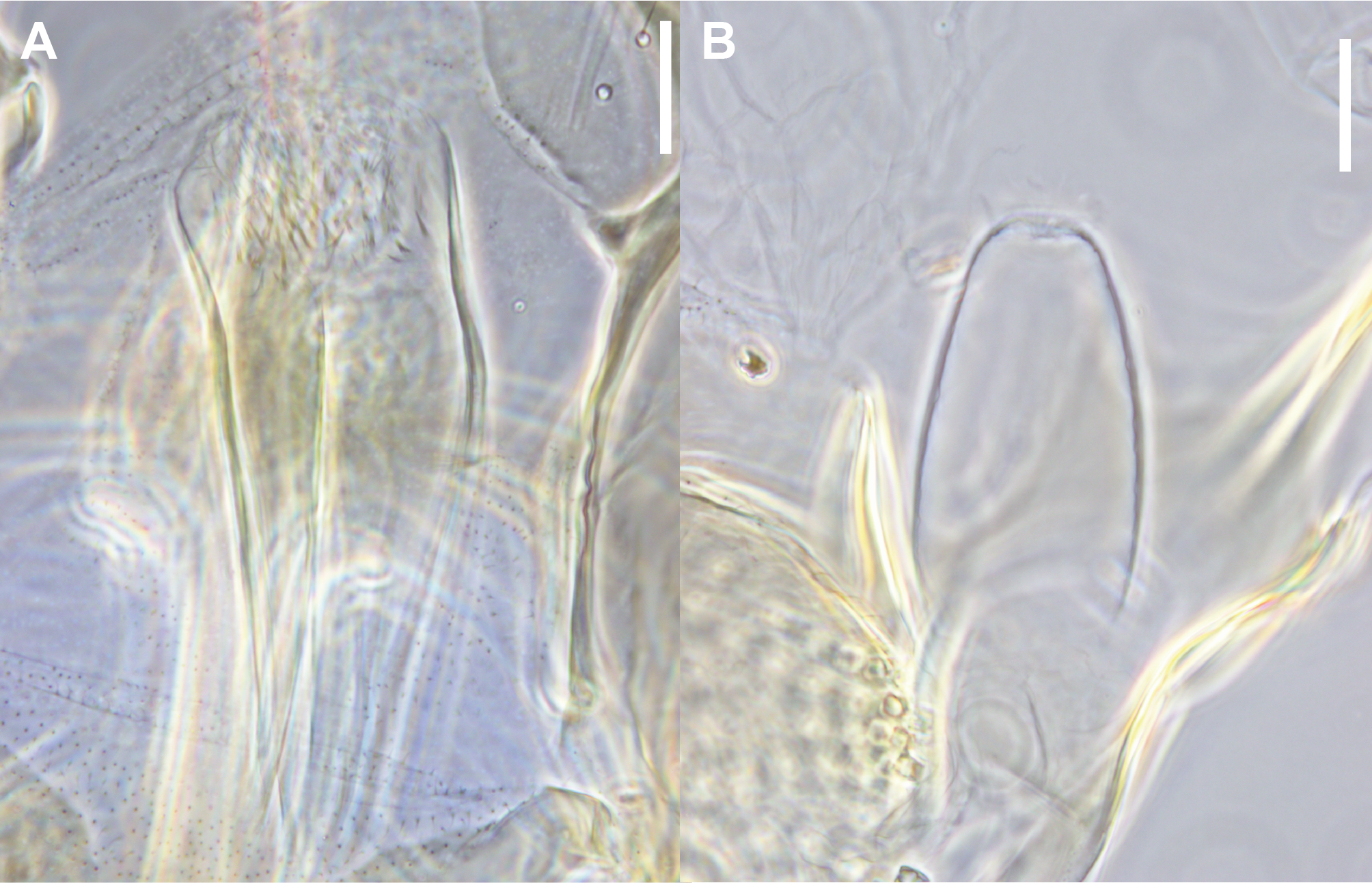

Supplement: Supplementary file 10 — Additional file 10: Fig. S10 Sergentomyia leechingae n. sp. female (voucher NEA0118.1) in phase contrast. A Pharynx, B spermathecae. Bars=20 µm. [file 13071_2025_7021_MOESM10_ESM.png]

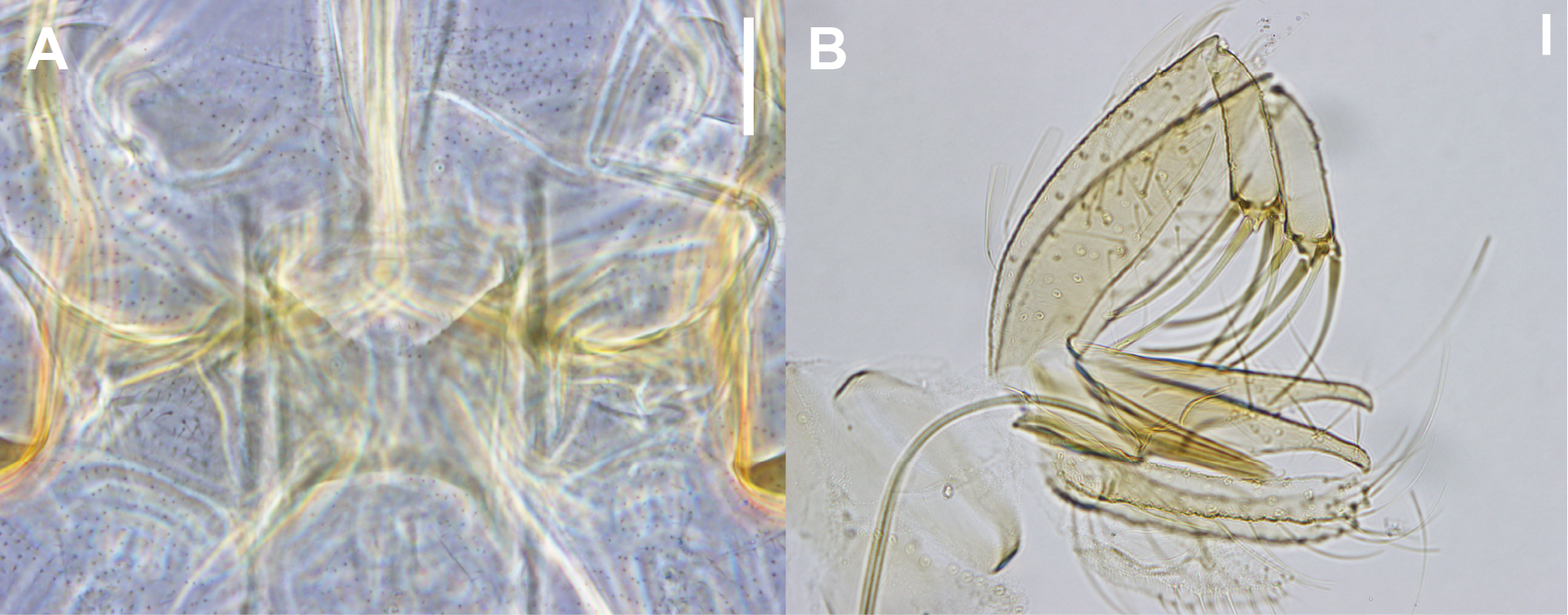

Supplement: Supplementary file 11 — Additional file 11: Fig. S11 Sergentomyia leechingae n. sp. male (voucher NEA0118.4). A Cibarium in phase contrast, B genitalia. Bars=20 µm. [file 13071_2025_7021_MOESM11_ESM.png]

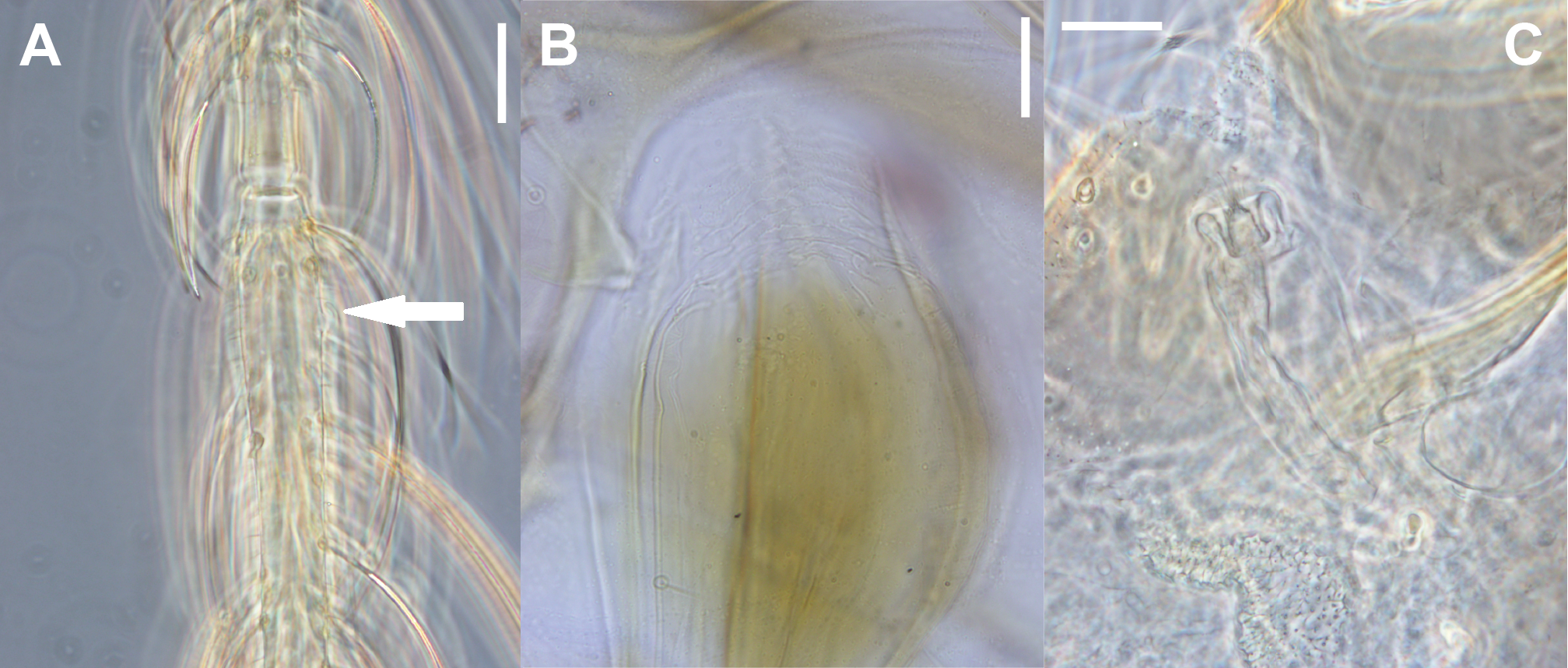

Supplement: Supplementary file 12 — Additional file 12: Fig. S12 Sergentomyia retrocalcarae n. sp. female (voucher NEA0120.1) in phase contrast. A 2nd flagellomere exhibiting the spur on the ascoid indicated by the arrow, B pharynx, C spermathecae. Bars=20 µm. [file 13071_2025_7021_MOESM12_ESM.png]

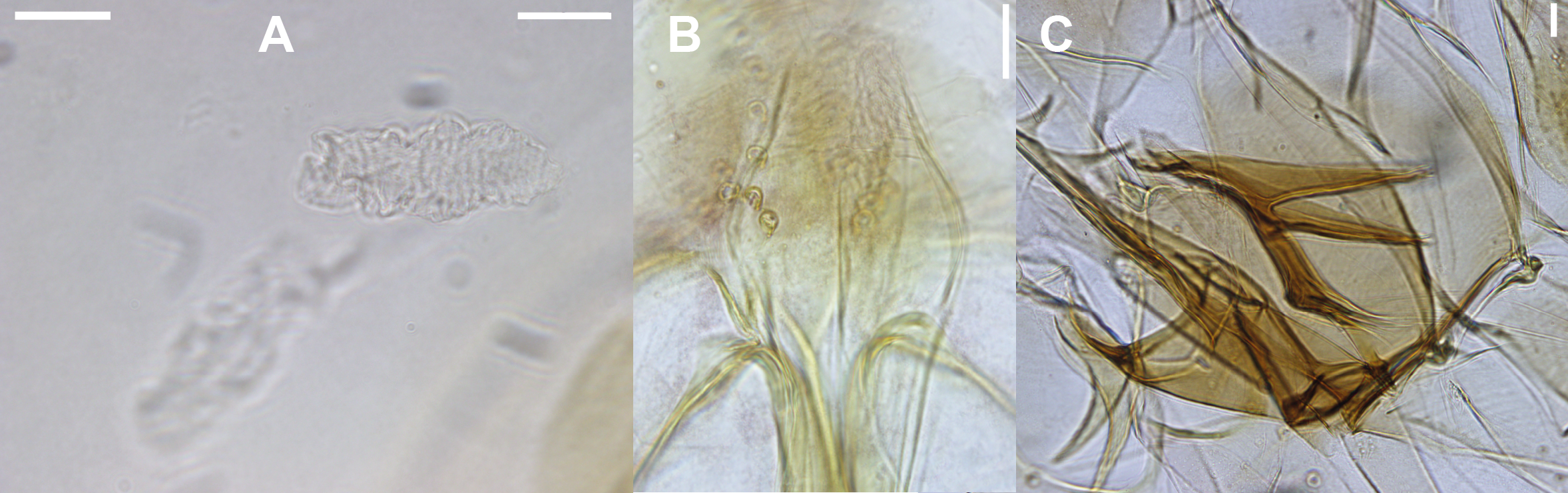

Supplement: Supplementary file 13 — Additional file 13: Fig. S13 Sergentomyia whartoni female (voucher NEA0069.1). A Spermathecae in phase contrast, B pharynx, D pigmented matafurca. Bars=20 µm. [file 13071_2025_7021_MOESM13_ESM.png]
