# Supplementary material for: Hidden in plain sight: discovery of sand flies in Singapore and description of four species new to science
Source: Parasit Vectors. 2025 Oct 9;18:402. doi: 10.1186/s13071-025-07021-5 (PMC12512794; doi:10.1186/s13071-025-07021-5)
Supplement: Supplementary file 14 — Additional file 14: Table S1 Mean interspecific genetic distances for cytb sequence pairs between five Phlebotomus (Euphlebotomus) species and Ph. (Anaphlebotomus) stantoni. Numbers (1-8) in the top row correspond to the species listed. Calculations were based on the p-distance model. Diagonal bold values indicate intraspecific mean distances. Names with a hashtag refer to specimens collected in Singapore (SG). [file 13071_2025_7021_MOESM14_ESM.docx]

**Additional file 14: Table S1** Mean interspecific genetic distances for *cytb* sequence pairs between five *Phlebotomus (Euphlebotomus)* species and *Ph. (Anaphlebotomus) stantoni*. Numbers (1-8) in the top row correspond to the species listed. Calculations were based on the p-distance model. Diagonal bold values indicate intraspecific mean distances. Names with a hashtag refer to specimens collected in Singapore (SG).

|  | Species | 1 | 2 | 3 | 4 | 5 | 6 | 7 | 8 |
| --- | --- | --- | --- | --- | --- | --- | --- | --- | --- |
| 1 | *Ph. seowpohi* n. sp.^#^ | **0.001** |  |  |  |  |  |  |  |
| 2 | *Ph. stantoni^#^* | 0.211 | **0.002** |  |  |  |  |  |  |
| 3 | *Ph. argentipes* | 0.125 | 0.197 | **0.014** |  |  |  |  |  |
| 4 | *Ph. barguesae* | 0.200 | 0.212 | 0.183 | **0.016** |  |  |  |  |
| 5 | *Ph. kiangsuensis* | 0.182 | 0.207 | 0.173 | 0.169 | **0.002** |  |  |  |
| 6 | *Ph. mascomai* | 0.159 | 0.171 | 0.143 | 0.117 | 0.141 | **0.010** |  |  |
| 7 | *Ph. seowpohi* n. sp. (Laos) | 0.010 | 0.213 | 0.125 | 0.202 | 0.180 | 0.161 | **0** |  |
| 8 | *Ph. stantoni* (non-SG) | 0.210 | 0.009 | 0.198 | 0.213 | 0.207 | 0.171 | 0.212 | **0.010** |
