# Supplementary material for: Hidden in plain sight: discovery of sand flies in Singapore and description of four species new to science
Source: Parasit Vectors. 2025 Oct 9;18:402. doi: 10.1186/s13071-025-07021-5 (PMC12512794; doi:10.1186/s13071-025-07021-5)
Supplement: Supplementary file 15 — Additional file 15: Table S2 Mean interspecific genetic distances for cytb sequence pairs between Sergentomyia species and Grassomyia species. Numbers (1-17) in the top row correspond to the species listed. Calculations were based on the p-distance model. Diagonal bold values indicate intraspecific mean distances. Names with a hashtag refer to specimens collected in Singapore (SG). NA denotes cases in which it was not possible to estimate genetic distances due to single DNA barcode. [file 13071_2025_7021_MOESM15_ESM.docx]

**Additional file 15: Table S2** Mean interspecific genetic distances for *cytb* sequence pairs between *Sergentomyia* species and *Grassomyia* species. Numbers (1-17) in the top row correspond to the species listed. Calculations were based on the p-distance model. Diagonal bold values indicate intraspecific mean distances. Names with a hashtag refer to specimens collected in Singapore (SG). NA denotes cases in which it was not possible to estimate genetic distances due to single DNA barcode.

|  | Species | 1 | 2 | 3 | 4 | 5 | 6 | 7 | 8 | 9 | 10 | 11 | 12 | 13 | 14 | 15 | 16 | 17 |
| --- | --- | --- | --- | --- | --- | --- | --- | --- | --- | --- | --- | --- | --- | --- | --- | --- | --- | --- |
| 1 | *Se. barraudi* grp^#^ | **0.033** |  |  |  |  |  |  |  |  |  |  |  |  |  |  |  |  |
| 2 | *Se. gubleri* n. sp.^#^ | 0.140 | **NA** |  |  |  |  |  |  |  |  |  |  |  |  |  |  |  |
| 3 | *Se. iyengari* grp^#^ | 0.109 | 0.123 | **0.001** |  |  |  |  |  |  |  |  |  |  |  |  |  |  |
| 4 | *Se. leechingae* n. sp.^#^ | 0.108 | 0.120 | 0.105 | **0** |  |  |  |  |  |  |  |  |  |  |  |  |  |
| 5 | *Se. retrocalcarae* n. sp.^#^ | 0.148 | 0.136 | 0.136 | 0.133 | **0.005** |  |  |  |  |  |  |  |  |  |  |  |  |
| 6 | *Se. whartoni^#^* | 0.131 | 0.120 | 0.096 | 0.132 | 0.124 | **NA** |  |  |  |  |  |  |  |  |  |  |  |
| 7 | *Grassomyia indica* | 0.143 | 0.147 | 0.129 | 0.142 | 0.139 | 0.113 | **0.001** |  |  |  |  |  |  |  |  |  |  |
| 8 | *Se. anodontis* | 0.119 | 0.114 | 0.094 | 0.094 | 0.149 | 0.125 | 0.134 | **0.005** |  |  |  |  |  |  |  |  |  |
| 9 | *Se. barraudi* grp | 0.057 | 0.125 | 0.117 | 0.091 | 0.157 | 0.134 | 0.143 | 0.104 | **0.044** |  |  |  |  |  |  |  |  |
| 10 | *Se. dvoraki* | 0.125 | 0.127 | 0.084 | 0.118 | 0.136 | 0.120 | 0.146 | 0.131 | 0.135 | **0.005** |  |  |  |  |  |  |  |
| 11 | *Se. gemmea* | 0.132 | 0.114 | 0.079 | 0.115 | 0.131 | 0.117 | 0.133 | 0.119 | 0.134 | 0.115 | **0.093** |  |  |  |  |  |  |
| 12 | *Se. hivernus* | 0.116 | 0.122 | 0.073 | 0.121 | 0.134 | 0.099 | 0.129 | 0.123 | 0.127 | 0.109 | 0.106 | **0.010** |  |  |  |  |  |
| 13 | *Se. khawi* | 0.127 | 0.099 | 0.050 | 0.103 | 0.142 | 0.106 | 0.130 | 0.102 | 0.122 | 0.106 | 0.052 | 0.088 | **0.004** |  |  |  |  |
| 14 | *Se. phadangensis* | 0.121 | 0.138 | 0.127 | 0.164 | 0.154 | 0.128 | 0.147 | 0.142 | 0.131 | 0.140 | 0.142 | 0.129 | 0.125 | **NA** |  |  |  |
| 15 | *Se. phasukae* | 0.131 | 0.132 | 0.119 | 0.132 | 0.146 | 0.097 | 0.132 | 0.139 | 0.130 | 0.140 | 0.125 | 0.126 | 0.111 | 0.142 | **0.035** |  |  |
| 16 | *Se. rudnicki* | 0.114 | 0.095 | 0.114 | 0.108 | 0.142 | 0.103 | 0.142 | 0.098 | 0.106 | 0.131 | 0.127 | 0.132 | 0.114 | 0.140 | 0.116 | **0.010** |  |
| 17 | *Se. raynali* | 0.117 | 0.140 | 0.121 | 0.119 | 0.116 | 0.119 | 0.122 | 0.126 | 0.128 | 0.134 | 0.117 | 0.127 | 0.118 | 0.152 | 0.114 | 0.104 | **NA** |
