# Supplementary material for: Hidden in plain sight: discovery of sand flies in Singapore and description of four species new to science
Source: Parasit Vectors. 2025 Oct 9;18:402. doi: 10.1186/s13071-025-07021-5 (PMC12512794; doi:10.1186/s13071-025-07021-5)
Supplement: Supplementary file 16 — Additional file 16: Table S3 Mean interspecific genetic distances for COI sequence pairs between four Phlebotomus (Euphlebotomus) species and Ph. (Anaphlebotomus) stantoni. Numbers (1-7) in the top row correspond to the species listed. Calculations were based on the p-distance model. Diagonal bold values indicate intraspecific mean distances. Names with a hashtag refer to specimens collected in Singapore (SG). NA denotes cases in which it was not possible to estimate genetic distances due to single DNA barcode. [file 13071_2025_7021_MOESM16_ESM.docx]

**Additional file 16: Table S3** Mean interspecific genetic distances for *COI* sequence pairs between four *Phlebotomus* (*Euphlebotomus*) species and *Ph.* (*Anaphlebotomus*) *stantoni*. Numbers (1-7) in the top row correspond to the species listed. Calculations were based on the p-distance model. Diagonal bold values indicate intraspecific mean distances. Names with a hashtag refer to specimens collected in Singapore (SG). NA denotes cases in which it was not possible to estimate genetic distances due to single DNA barcode.

|  | Species | 1 | 2 | 3 | 4 | 5 | 6 | 7 |
| --- | --- | --- | --- | --- | --- | --- | --- | --- |
| 1 | *Ph. seowpohi* n. sp.^#^ | **0.002** |  |  |  |  |  |  |
| 2 | *Ph. stantoni^#^* | 0.162 | **0.010** |  |  |  |  |  |
| 3 | *Ph. argentipes* | 0.146 | 0.154 | **0.009** |  |  |  |  |
| 4 | *Ph. barguesae* | 0.169 | 0.157 | 0.140 | **0.054** |  |  |  |
| 5 | *Ph. mascomai* | 0.160 | 0.171 | 0.144 | 0.138 | **0.011** |  |  |
| 6 | *Ph. seowpohi* n. sp. (Laos) | 0.022 | 0.163 | 0.141 | 0.171 | 0.163 | **NA** |  |
| 7 | *Ph. stantoni* (non-SG) | 0.163 | 0.049 | 0.150 | 0.154 | 0.161 | 0.161 | **0.010** |
