# Supplementary material for: Hidden in plain sight: discovery of sand flies in Singapore and description of four species new to science
Source: Parasit Vectors. 2025 Oct 9;18:402. doi: 10.1186/s13071-025-07021-5 (PMC12512794; doi:10.1186/s13071-025-07021-5)
Supplement: Supplementary file 17 — Additional file 17: Table S4 Mean interspecific genetic distances for COI sequence pairs between Sergentomyia species. Numbers (1-17) in the top row correspond to the species listed. Calculations were based on the p-distance model. Diagonal bold values indicate intraspecific mean distances. Names with a hashtag refer to specimens collected in Singapore (SG). NA denotes cases in which it was not possible to estimate genetic distances due to single DNA barcode. [file 13071_2025_7021_MOESM17_ESM.docx]

**Additional file 17: Table S4** Mean interspecific genetic distances for *COI* sequence pairs between *Sergentomyia* species. Numbers (1-17) in the top row correspond to the species listed. Calculations were based on the p-distance model. Diagonal bold values indicate intraspecific mean distances. Names with a hashtag refer to specimens collected in Singapore (SG). NA denotes cases in which it was not possible to estimate genetic distances due to single DNA barcode.

|  | Species | 1 | 2 | 3 | 4 | 5 | 6 | 7 | 8 | 9 | 10 | 11 | 12 | 13 | 14 | 15 | 16 | 17 |
| --- | --- | --- | --- | --- | --- | --- | --- | --- | --- | --- | --- | --- | --- | --- | --- | --- | --- | --- |
| 1 | *Se. barraudi* grp^#^ | **0.027** |  |  |  |  |  |  |  |  |  |  |  |  |  |  |  |  |
| 2 | *Se. gubleri* n. sp.^#^ | 0.155 | **NA** |  |  |  |  |  |  |  |  |  |  |  |  |  |  |  |
| 3 | *Se. iyengari* grp^#^ | 0.121 | 0.154 | **0.001** |  |  |  |  |  |  |  |  |  |  |  |  |  |  |
| 4 | *Se. leechingae* n. sp.^#^ | 0.104 | 0.155 | 0.131 | **0** |  |  |  |  |  |  |  |  |  |  |  |  |  |
| 5 | *Se. retrocalcarae* n. sp.^#^ | 0.150 | 0.126 | 0.145 | 0.165 | **0.001** |  |  |  |  |  |  |  |  |  |  |  |  |
| 6 | *Se. whartoni*^#^ | 0.125 | 0.138 | 0.150 | 0.125 | 0.155 | **NA** |  |  |  |  |  |  |  |  |  |  |  |
| 7 | *Se. anodontis* | 0.115 | 0.118 | 0.131 | 0.133 | 0.125 | 0.128 | **0.073** |  |  |  |  |  |  |  |  |  |  |
| 8 | *Se. babu* | 0.099 | 0.148 | 0.128 | 0.031 | 0.155 | 0.122 | 0.125 | **0.019** |  |  |  |  |  |  |  |  |  |
| 9 | *Se. baghdadis* | 0.117 | 0.149 | 0.125 | 0.125 | 0.144 | 0.156 | 0.140 | 0.124 | **0.125** |  |  |  |  |  |  |  |  |
| 10 | *Se. barraudi* (non-SG) | 0.068 | 0.152 | 0.129 | 0.103 | 0.155 | 0.136 | 0.127 | 0.101 | 0.122 | **0.073** |  |  |  |  |  |  |  |
| 11 | *Se. gemmea* | 0.128 | 0.165 | 0.098 | 0.154 | 0.157 | 0.147 | 0.134 | 0.149 | 0.140 | 0.143 | **0.014** |  |  |  |  |  |  |
| 12 | *Se. hivernus* | 0.116 | 0.139 | 0.097 | 0.131 | 0.136 | 0.127 | 0.124 | 0.130 | 0.126 | 0.125 | 0.120 | **0.007** |  |  |  |  |  |
| 13 | *Se. insularis* | 0.099 | 0.150 | 0.132 | 0.043 | 0.154 | 0.116 | 0.128 | 0.027 | 0.128 | 0.103 | 0.143 | 0.126 | **0.019** |  |  |  |  |
| 14 | *Se. iyengari* (non-SG) | 0.118 | 0.144 | 0.098 | 0.135 | 0.137 | 0.131 | 0.127 | 0.133 | 0.128 | 0.128 | 0.126 | 0.013 | 0.129 | **0.019** |  |  |  |
| 15 | *Se. khawi* | 0.128 | 0.162 | 0.095 | 0.153 | 0.155 | 0.147 | 0.132 | 0.148 | 0.139 | 0.140 | 0.012 | 0.119 | 0.144 | 0.125 | **0.014** |  |  |
| 16 | *Se. perturbans* | 0.125 | 0.132 | 0.147 | 0.128 | 0.151 | 0.022 | 0.124 | 0.125 | 0.155 | 0.136 | 0.146 | 0.124 | 0.123 | 0.127 | 0.146 | **0.030** |  |
| 17 | *Se. rudnicki* | 0.137 | 0.148 | 0.141 | 0.137 | 0.146 | 0.128 | 0.138 | 0.141 | 0.142 | 0.148 | 0.160 | 0.134 | 0.140 | 0.134 | 0.158 | 0.130 | **0.002** |
