# Supplementary material for: Hidden in plain sight: discovery of sand flies in Singapore and description of four species new to science
Source: Parasit Vectors. 2025 Oct 9;18:402. doi: 10.1186/s13071-025-07021-5 (PMC12512794; doi:10.1186/s13071-025-07021-5)
Supplement: Supplementary file 18 — Additional file 18: Table S5 Morphometric measurements (in µm) of Phlebotomus seowpohi n. sp. specimens. Values are presented as mean (minimum-maximum). [file 13071_2025_7021_MOESM18_ESM.docx]

**Additional file 18: Table S5** Morphometric measurements (in µm) of *Phlebotomus seowpohi* n. sp. specimens. Values are presented as mean (minimum-maximum).

|  | | **Females (n = 5)** | |  | **Males (n = 4)** | |
| --- | --- | --- | --- | --- | --- | --- |
| **HEAD** | | | | | | |
|  | length | 407 | (360-432) |  | 470.25 | (351-774) |
|  | width | 368.4 | (354-380) |  | 429.5 | (305-721) |
| Clypeus | | | | | | |
|  | Length | 142.4 | (109-161) |  | 139.75 | (100-232) |
|  | Width | 58.8 | (54-63) |  | 62.5 | (47-87) |
| Eyes | | | | | | |
|  | Length | 255.2 | (242-273) |  | 244.75 | (239-252) |
|  | Width | 138.2 | (119-153) |  | 131.5 | (128-140) |
| Flagellomeres | | | | | | |
|  | f1 | 237.6 | (224-256) |  | 235.75 | (218-262) |
|  | f2 | 100.8 | (97-105) |  | 103.39 | (100-109) |
|  | length ascoid f2 | 72.29 | (66.87-74.80) |  | 63.53 | (55.16-70.04) |
|  | ascoïd f2/f2 | 0.72 | (0.66-0.76) |  | 0.61 | (0.55-0.66) |
|  | f3 | 104.4 | (100-111) |  | 105.25 | (101-113) |
|  | f12 | 87 | (82-92) |  | 80.75 | (77-84) |
|  | f13 | 83.6 | (74-93) |  | 71.5 | (70-73) |
|  | f14 | 79.2 | (71-84) |  | 72.75 | (68-78) |
|  | f2+f3 | 205.2 | |  | 212 | |
|  | antennal formula | 2/f1-f13 | |  | 2/f1-f13 | |
| Palpi | | | | | | |
|  | p1 | 44.4 | (40-49) |  | 41.75 | (30-53) |
|  | p2 | 96.4 | (91-101) |  | 76.75 | (68-86) |
|  | p3 | 138.8 | (130-149) |  | 120.75 | (116-125) |
|  | p4 | 66.4 | (63-69) |  | 57 | (52-59) |
|  | p5 | 171 | (160-185) |  | 159.5 | (147-169) |
|  | palpal formula | 1. 4. 2. 3. 5 | |  | 1. 4. 2. 3. 5 | |
| Labrum | | | | | | |
|  | length | 254.6 | (227-265) |  | 193 | (182-211) |
|  |  |  |  |  |  |  |
| **WING** | | | | | | |
|  | Length | 1905.4 | (1786-2040) |  | 1719.25 | (1659-1800) |
|  | width | 657.2 | (622-712) |  | 560.5 | (514-602) |
|  | R5 | 1198 | (1127-1298) |  | 1044.25 | (992-1120) |
|  | alpha | 550.6 | (502-694) |  | 405.75 | (381-427) |
|  | beta | 292.6 | (207-571) |  | 305.25 | (169-558) |
|  | gamma | 213.2 | (170-287) |  | 233.25 | (167-367) |
|  | delta | 96 | (69-122) |  | 42.25 | (8-79) |
|  | pi | 79.2 | (52-148) |  | 96.75 | (55-167) |
|  | Epsilon | 635.2 | (549-692) |  | 546.75 | (529-563) |
|  | w/gamma | 3.08 | |  | 2.40 | |
|  |  |  | |  |  | |
| **GENITALIA** | | | | | | |
|  | Gonostyle |  |  |  | 161 | (154-169) |
|  | Paramere 1 |  |  |  | 159 | (147-182) |
|  | Paramere 2 |  |  |  | 33.25 | (28-43) |
|  | Paramere 3 |  |  |  | 22.75 | (20-28) |
|  | Paramere Sheath |  |  |  | 106.25 | (69-166) |
| Epandrial Lobes | | | | | | |
|  | Length |  |  |  | 291.25 | (249-330) |
|  | Width |  |  |  | 29.25 | (25-36) |
| Sperm pump | | | | | | |
|  | Length |  |  |  | 126.5 | (123-132) |
|  | Width |  |  |  | 24 | (20-29) |
| Ejaculatory apodeme | | | | | | |
|  | Length |  |  |  | 97.75 | (92-104) |
|  | Width |  |  |  | 17.75 | (10-25) |
| Aedeagal ducts | | | | | | |
|  | Length |  |  |  | 263.75 | (244-282) |
| Cercus | | | | | | |
|  | Length | 122.6 | (81-137) |  |  |  |
|  | Width | 51.8 | (23-76) |  |  |  |
| Spermathecae | | | | | | |
|  | Length | 33.6 | (23-43) |  |  |  |
|  | Width | 11.2 | (9-14) |  |  |  |
| Spermathecal individual duct | | | | | | |
|  | Length | 53.67 | (43-67) |  |  |  |
| Spermathecal common duct | | | | | | |
|  | Length | 69 | (59-84) |  |  |  |
